# Properties of Metal Hydrides of the Iron Triad

**DOI:** 10.1021/jacs.3c08925

**Published:** 2023-12-07

**Authors:** Arie J. H. Multem, Guilherme L. Tripodi, Jana Roithová

**Affiliations:** Department of Spectroscopy and Catalysis, Institute for Molecules and Materials, Radboud University Nijmegen, Heyendaalseweg 135, 6525 AJ Nijmegen, The Netherlands

## Abstract

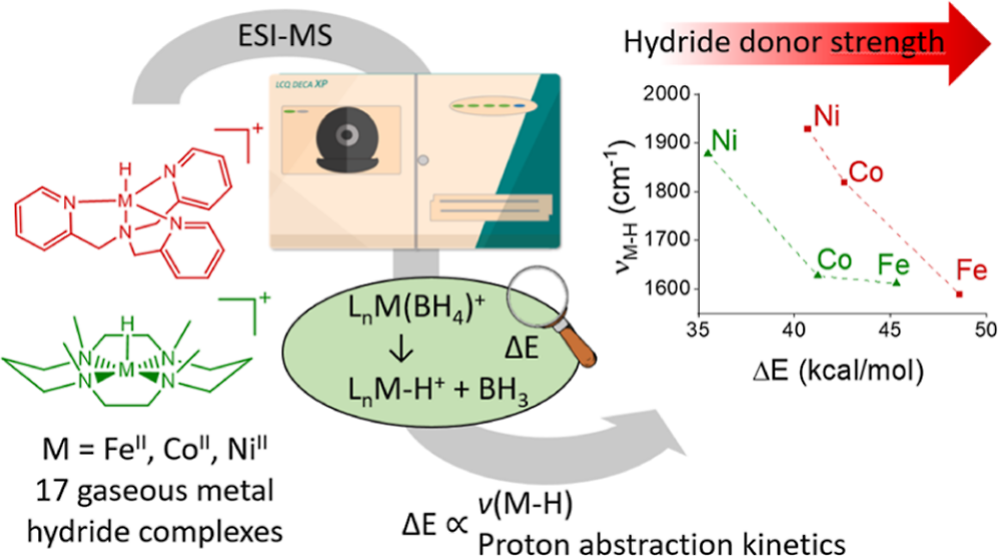

Metal hydride complexes are essential intermediates in hydrogenation reactions. The hydride-donor ability determines the scope of use of these complexes. We present a new, simple mass-spectrometry method to study the hydride-donor ability of metal hydrides using a series of 18 iron, cobalt, and nickel complexes with *N-* and *P-*based ligands (L). The mixing of [(L)M^II^(OTf)_2_] with NaBH_4_ forms [(L)M^II^(BH_4_)]^+^ (M = Fe, Co, Ni) that can be detected by electrospray ionization mass spectrometry. Energy-resolved collision-induced dissociations of [(L)M^II^(BH_4_)]^+^ provide threshold energies (Δ*E*_CID_) for the formations of [(L)M^II^(H)]^+^ that correlate well with the hydride donor ability of the metal hydride complexes. We studied the vibrational and electronic spectra of the generated metal hydrides, assigned their structure and spin state, and demonstrated a good correlation between Δ*E*_CID_ and the M–H stretching vibration frequencies. The Δ*E*_CID_ also correlates with reaction rates for hydride transfer reactivity in the gas phase and known reactivity trends in the solution phase.

## Introduction

**Chart 1:**
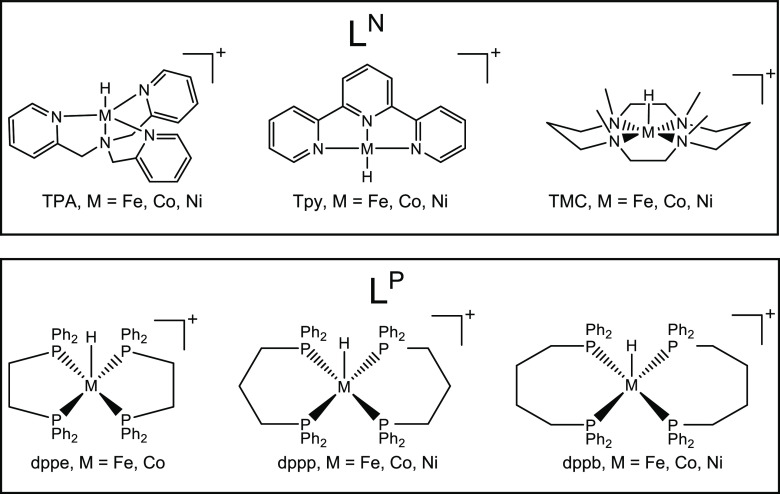
Overview and Nomenclature of the Investigated Complexes. Nitrogen-Based Ligands L^N^ (Top) with Fe, Co, and Ni and Phosphorus-Based Ligands L^P^ (Bottom) with Fe and Co

Metal-based homogeneous hydrogenation has been used in the last decades for efficient and selective reduction of C=O, C=N, and C=C bonds to produce alcohols, amines, and alkanes, respectively.^1^ The advance in homogeneous catalysis has mainly focused on noble metals.^2^ Especially rhodium-, ruthenium-, and iridium-based catalysts are well-known for their high efficiency and selectivity under mild conditions.^2,3^ However, noble metals are rare, expensive, and generally more toxic than their first-row analogues.^4^ Therefore, alternative hydrogenation catalysts based on first-row transition metals have been developed in the past decade.^5^ Metal catalysts capable of hydrogenating CO_2_ to formic acid or methanol under mild conditions would be particularly exciting.^6^ Detailed explorations of the hydrogenation mechanism with ruthenium-based catalysts revealed the key role of the metal hydride intermediates.^7^ Similarly, iron, cobalt, and nickel hydrides have been identified as the key intermediates in many catalytic cycles involving hydrogenation reactions.^5b^ For example, Fe(II) hydrides are capable of hydrogenating C=O,^8^ C=N,^9^ and C=C^10^ bonds with moderate activity. The Co(II)^11^ and Ni(II)^12^ hydrides show similar reactivities. Mass spectrometry is a helpful technique for studying highly reactive and short-lived charged species that can be transferred to the gas phase via electrospray ionization.^13^ Advanced mass spectrometry techniques allow measurements of vibrational and electronic spectra of the mass-selected ions^14^ and their thermochemical properties.^15^ Here, we investigate bond dissociation energies, ion–molecule reactions, and infrared photodissociation spectra of isolated cationic metal hydrides in the gas phase (Chart 1). In solution, metal hydride properties, such as their hydride-donor power, can be severely affected by solvation effects.^16^ Studying the metal hydrides in the gas phase avoids the interferences present in the solution and thus allows us to perform structure–reactivity correlations confidently.

## Results

### Generation of the Hydride Complexes

Metal hydride complexes were generated by the reaction between [(L)M^II^(OTf)_2_] and NaBH_4_ in a flow reactor coupled to the electrospray ionization source of a triple quadrupole mass spectrometer (Supporting Information, Figure S2). Primarily, we form metal borohydride complexes in flow and transfer them to the gas phase by electrospray. In the gas phase, the borohydrides fragment by collision-induced dissociation (CID) to form the desired metal hydrides (reaction 1).
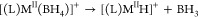
1

**Figure 1:**
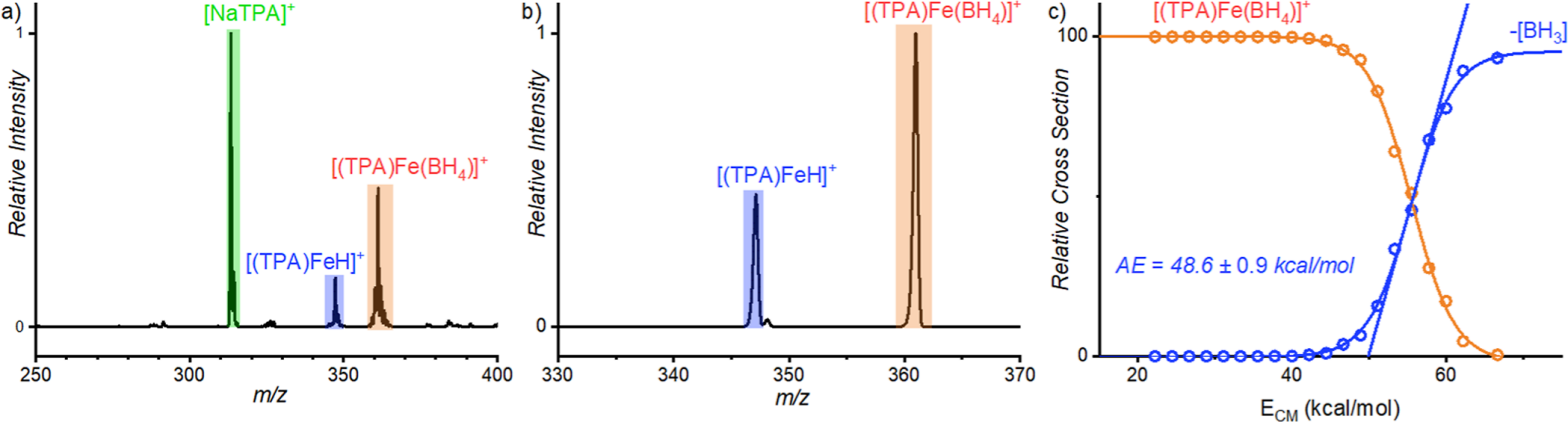
(a) ESI-MS spectrum of a mixture of iron complex and sodium borohydride. (b) Collision-induced dissociation (CID) spectrum of mass-selected [(TPA)Fe(BH_4_)]^+^ at *E*_coll_ = 54 kcal/mol. (c) Breakdown diagram of [(TPA)Fe(BH_4_)]^+^; the dots are experimental data, and the lines are fitted sigmoid functions. Linear extrapolation gives us the appearance energy (AE) or the bond dissociation energy (BDE) of the fragmentation.

**Scheme 1:**
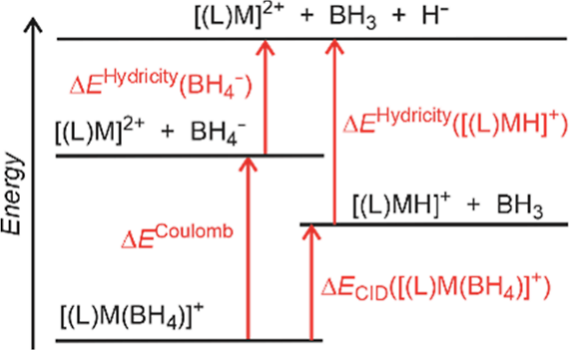
Δ*E*_CID_ Relationship to Complex Hydricity

To assess the metal hydride donor strength, we quantified the required energy for the fragmentation (Δ*E*_CID_) using energy-resolved CID experiments (Figure 1). The determined Δ*E*_CID_ relates to the hydricity (free energy of an M–H bond cleavage to generate H^–^) of a metal hydride complex according to eq 2
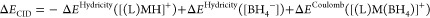
2where Δ*E*^Coulomb^[(L)M(BH_4_)]^+^ is the free energy for the dissociation of [(L)M(BH_4_)]^+^ into [(L)M]^2+^ and (BH_4_)^−^ (Scheme 1).

This energy will be dominated by the Coulomb interaction between a dication and an anion. However, ligand L plays a role in stabilizing the charge at the metal center. The charge stabilization according to L plays a much larger role than the metal identity (Tables S7 and S8). Hence, we assume Δ*E*^Coulomb^([(L)M(BH_4_)]^+^) as a constant for a series of Fe, Co, and Ni complexes with the same ligand L. Δ*E*^Hydricity^([BH_4_]^−^) is also a constant. Therefore, the determined Δ*E*_CID_ is related to the gas-phase thermodynamic hydricity in a series of [(L)MH]^+^ complexes with the same ligand L, according to eq 3
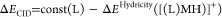
3

### Bond Dissociation Energies

**Figure 2:**
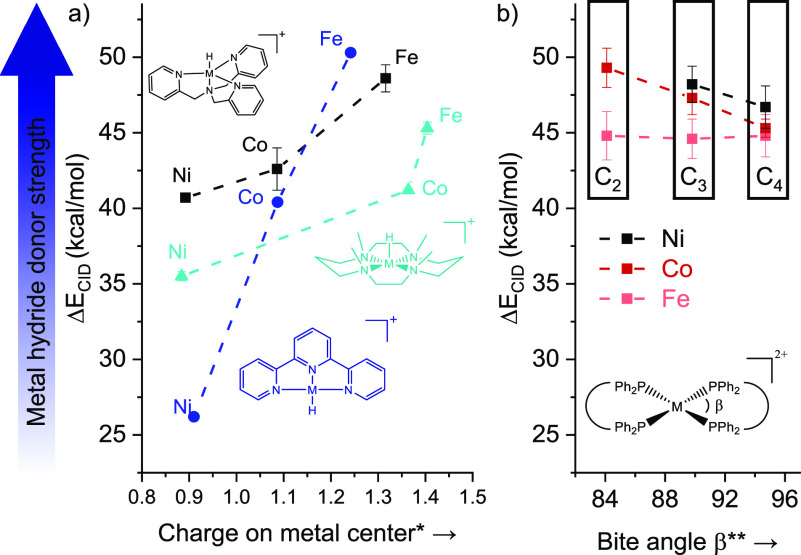
Experimental bond dissociation energies (Δ*E*_CID_) of the studied metal hydride complexes (Chart 1) for the loss of BH_3_ from [(L)M(BH_4_)]^+^ generating the metal hydride ion. (a) Effect of the charge on the metal center on Δ*E*_CID_. (b) Effect of the bite angle. *Calculated with the natural bond orbital (NBO) analysis at B3LYP-D3/6-311++G(d,p)//B3LYP/6-311G(d,p). **Calculated at B3LYP-D3/6-311++G(d,p).

We have studied bond dissociation energies of a series of complexes with the general composition [(L)_n_M(BH_4_)]^+^ with polydentate nitrogen- and phosphorus-based ligands (L^N^ and L^P^, Chart 1). Results for the complexes with the *N-*based ligands TPA, TMC, and Tpy show that the Δ*E*_CID_ energies for forming the respective metal hydrides decrease by 5–10 kcal/mol when changing the metal center in the order Fe → Co → Ni (Table 1). The M–H bond energy should correlate with the orbital overlap between the metal center and the hydride. The larger the overlap, the greater the electron-density delocalization from hydride to metal. Hence, the M–H bond energy should correlate with the charge density at the metal center. This trend is indeed observed (Figure 2a). The coordination environment around the metal can have a dramatic effect, as exemplified by the L^N^-nickel complexes exhibiting Δ*E*_CID_s that span from 26 to 41 kcal mol^–1^. In contrast, the Δ*E*_CID_ values for the L^P^-complexes are rather similar and do not follow the Fe > Co > Ni trend.
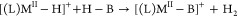
4

**Table 1:** Experimental Bond Dissociation Energies (Δ*E*_CID_) and Metal Hydride Vibrations (*v*(M–H)) of the Investigated Complexes

N-based ligands(spin multiplicity)	Δ*E*_CID_ (kcal/mol)	ν M–H (cm^–1^)	P-based ligands	Δ*E*_CID_ (kcal/mol)
[(TPA)FeH]^+^ (*S* = 2)	48.6 ± 0.9	1588	[(dmpe)_2_FeH]^+^	46.4 ± 1.2
[(TPA)CoH]^+^ (*S* = 1/2)	42.6 ± 1.4	1819	[(dppe)_2_FeH]^+^	44.8 ± 1.6
[(TPA)NiH]^+^ (*S* = 0)	40.7 ± 0.08	1929	[(dppe)_2_CoH]^+^	49.3 ± 1.3
[(TMC)FeH]^+^ (*S* = 2)	45.3 ± 0.4	1611	[(dppp)_2_FeH]^+^	44.6 ± 1.3
[(TMC)CoH]^+^ (*S* = 3/2)	41.2 ± 0.1	1627	[(dppp)_2_CoH]^+^	47.3 ± 1.1
[(TMC)NiH]^+^ (*S* = 0)	35.5 ± 0.3	1878	[(dppp)_2_NiH]^+^	48.2 ± 1.2
[(Tpy)FeH]^+^ (*S* = 2)	50.3 ± 0.1	1688	[(dppb)_2_FeH]^+^	44.8 ± 1.4
[(Tpy)CoH]^+^ (*S* = 1/2)	40.4 ± 0.2	1843	[(dppb)_2_CoH]^+^	45.3 ± 0.6
[(Tpy)NiH]^+^ (*S* = 0)	26.2 ± 0.2	1915	[(dppb)_2_NiH]^+^	46.7 ± 1.4

The hydride donor ability of the L^P^-complexes can be modulated by the electronic properties of the phosphine ligand and their natural bite angle. For instance, an increase of the σ-donor properties of the dppe ligand by phenyl-to-methyl substitution at the phosphorus atom leads to the complex [(dmpe)_2_Fe(BH_4_)]^+^ having a larger Δ*E*_CID_ than [(dppe)_2_Fe(BH_4_)]^+^ by 1.6 kcal mol^–1^. Similarly, decreasing the L^P^ natural bite angle increases the Δ*E*_CID_ of Co and Ni complexes by ca. 2 kcal mol^–1^.

### Infrared and Visible Photodissociation Spectroscopy

**Figure 3:**
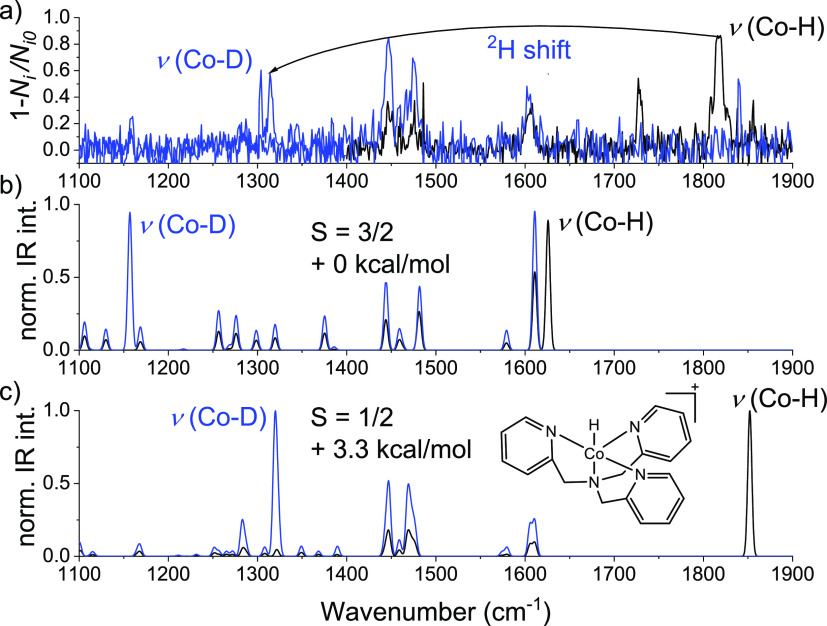
(a) Neon-tagging IRPD spectrum of [(TPA)CoH]^+^, ^2^H isotopologue spectrum in blue. (b,c) B3LYP-D3/6-311++G** theoretical IRPD spectra of the quartet (b) and doublet (c) spin states, ^2^H isotopologue spectrum in blue (scaling factor is 0.98).

**Figure 4:**
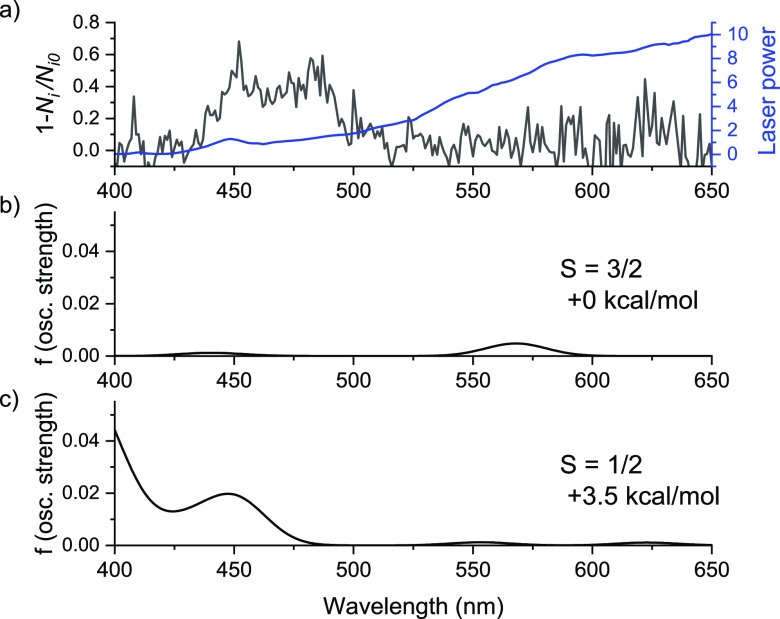
(a) Neon-tagging visPD spectrum of [(TPA)CoH]^+^, and the right axis refers to the wavelength-power in blue. (b,c) B3LYP-D3/6-311++G** theoretical visPD spectra of the quartet (b) and doublet (c) spin states.

The metal hydride bonds can be characterized by their stretching frequencies. To this end, we measured helium-tagging infrared photodissociation spectra of the isolated hydrides [(L^N^)MH]^+^ and deuterated analogues [(L^N^)MD]^+^. The metal-hydride stretching vibration was identified by the isotopic shift of the M–H band upon deuteration. The *ν*(M–H) frequencies were found in the 1550–1950 cm^–1^ range (Table 1). We interpret the IR spectra based on the comparison with DFT (B3LYP/6-311++G**) calculated spectra of multiple isomers of the metal hydride complexes having different spin states (Figures 3 and S3–S11). DFT predicts that the spin configuration of the metal center significantly affects the M–H vibration (Figures S3–S11). The high spin state configuration of the metal complex shifts the metal hydride vibration to a lower wavenumber and thus to a weaker hydride bond strength (Table 1). All experimental spectra match with the theoretical IR spectra of the most stable metal hydride isomers in the ground electronic state except [(TPA)CoH]^+^, [(TMC)NiH]^+^, and [(Tpy)FeH]^+^. For example, the calculated ground state of [(TPA)CoH]^+^ is the quartet state. Still, the infrared photodissociation (IRPD) spectrum better matches the theoretical spectrum of the doublet state complex that is predicted to be 3.3 kcal/mol higher in energy (compare the position of the Co–H band in Figure 3). To confirm the spin state, we measured the electronic spectrum of [(TPA)CoH]^+^ using neon-tagging visible photodissociation (visPD) spectroscopy (Figure 4). The visPD spectrum of [(TPA)CoH]^+^ shows a band at 480 nm that agrees with the DFT-computed spectrum of the doublet state complex. The B3LYP density functional can overstabilize the high-spin states.^17^

**Figure 5:**
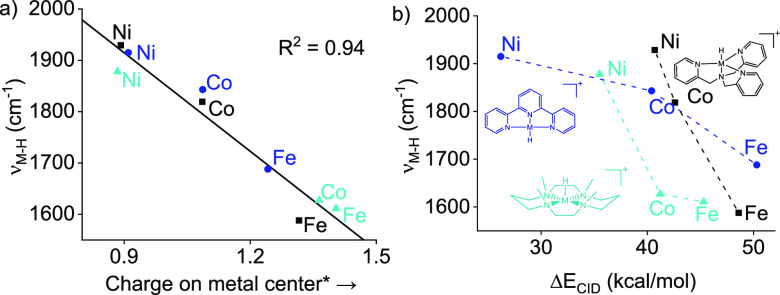
(a) Linear regression of the experimentally obtained *v*(M–H) (Table 1) versus the calculated charge localization on the metal center of [(L)M–H]^+^ (the colors refer to different ligands shown in (b). *Calculated with NBO analysis (B3LYP-D3/6-311++G(d,p)//B3LYP/6-311G(d,p)). (b) Plot of the metal hydride vibrations in cm^–1^ versus the experimental bond dissociation energies Δ*E*_CID_ in kcal/mol.

Here, B3LYP predicts the quartet spin state as the ground state, while the experiment shows that the doublet state is the ground state of the complex. Hence, even though the B3LYP calculations predict a wrong ground state, the calculated IR and vis spectra allow us to assign the isolated complexes’ spin states correctly. All studied iron hydride complexes have a high spin configuration (*S* = 2), whereas all nickel complexes have a low spin state (*S* = 0). Cobalt hydride complexes can adopt both the high spin (*S* = 3/2) and the low spin (*S* = 1/2) state configurations, depending on the ligand. The identified M–H bands are isolated, suggesting negligible coupling with other vibrational modes. Hence, the stretching frequency should directly correlate with the M–H bond strength and thus represent a thermodynamic measure of the hydricity in the gas phase. Accordingly, we found an excellent correlation between the metal hydride frequency and the DFT Mulliken charges on the metal center of the metal hydride species (Figure 5a).

The increasing electron delocalization from the hydride to the metal makes the M–H bond more covalent, which increases the bond strength and is reflected in the increased metal-hydride stretching frequency. Gratifyingly, the trends observed for the M–H stretching frequencies match those in the CID experiments (Figures 2a and 5b). While IRPD spectroscopy is laborious and not always available, CID experiments are common. Through this correlation, we demonstrate that CID experiments can be taken as a relative measure of the hydricity of gaseous metal complexes.

### Reactions in the Gas Phase

The determined hydricities of the complexes should correlate with the hydride-donating reactivities in the bimolecular reactions. We have explored this reactivity with different neutral reactants (Figures S52–S60) and found that we can observe H_2_ formation in the reactions of the metal hydrides with thiophenol and formic acid (reaction 4, Figure 7). We also tested insertion reactions with unsaturated compounds, but the observed reactivity was insufficient for comparing different complexes. Most likely, the insertion reactions proceed via higher energy barriers and are thus unaccessible in the gas phase (see also Figure S61).

**Figure 6:**
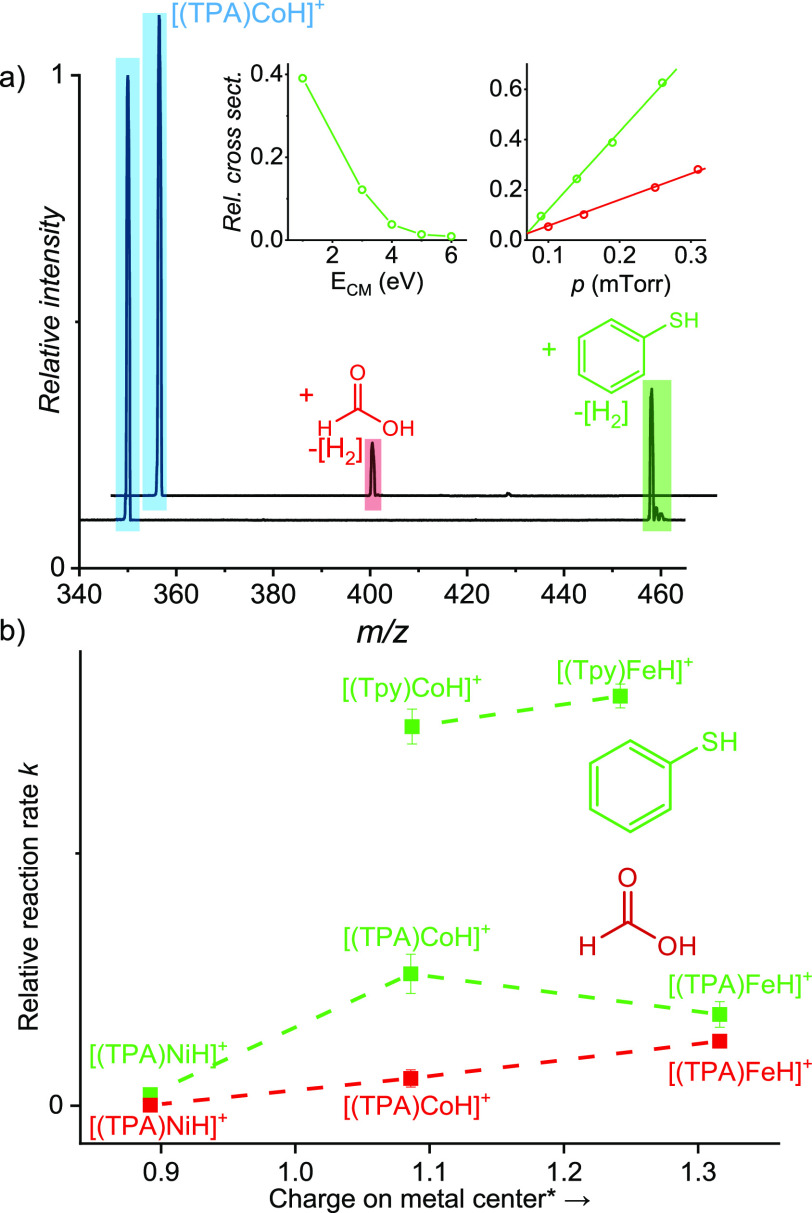
(a) ESI-MS spectrum of mass-selected ion, [(TPA)CoH]^+^, reacting with thiophenol (green) and formic acid (red) at *p* = 0.31 mTorr. (b) Gas-phase kinetics of the bimolecular reaction of the metal hydride complexes having the TPA or Tpy ligands with thiophenol (green) and formic acid (red).

**Figure 7:**
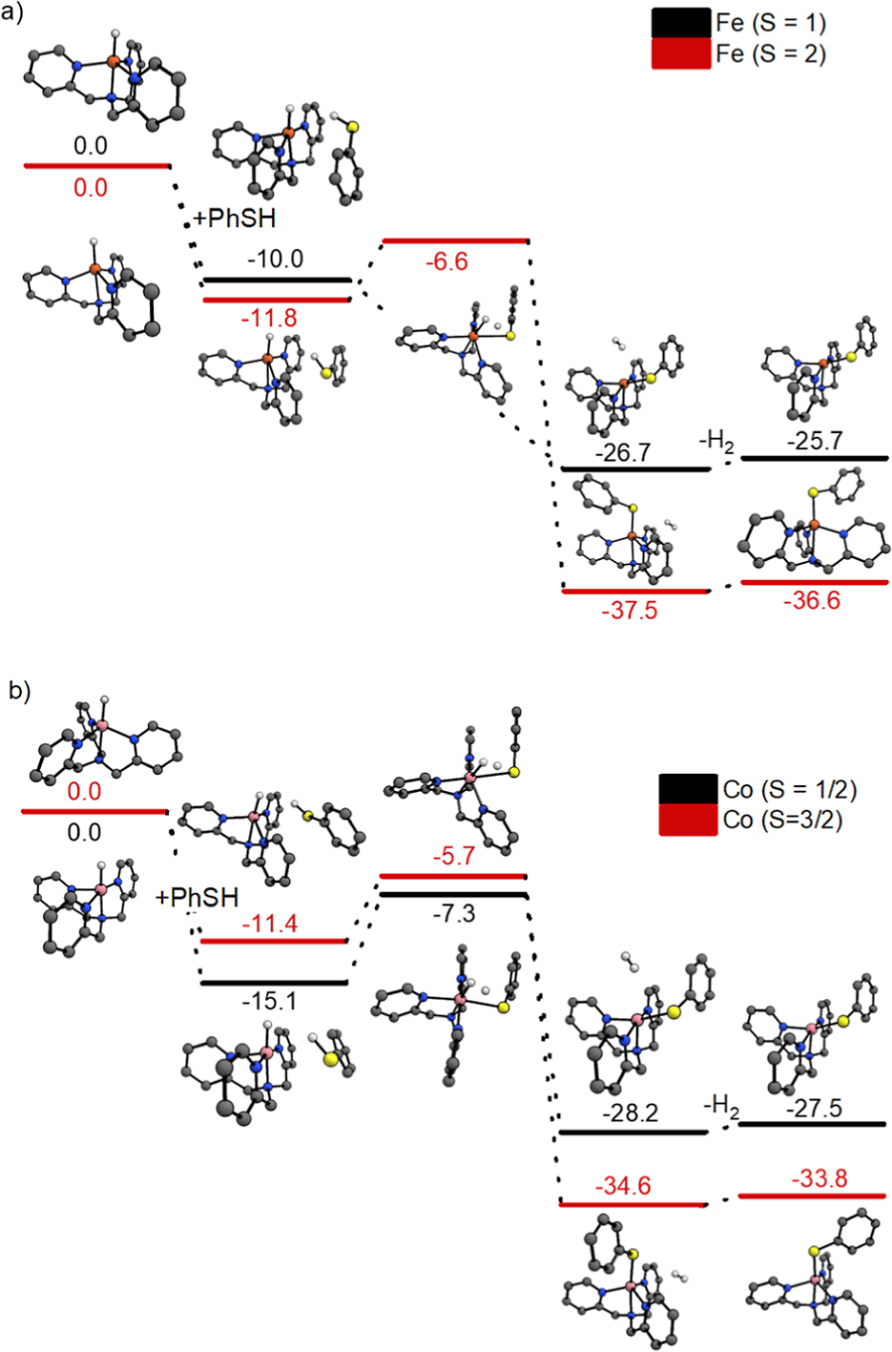
(a) Potential energy surface (ΔΔ*H*^0K^, in kcal/mol, B3LYP-D3/6-311++G(d,p)) of [(TPA)FeH]^+^ with thiophenol for the *S* = 1 (black) and the *S* = 2 spin state (red). (b) Potential energy surface (ΔΔ*H*^0K^, in kcal/mol, B3LYP-D3/6-311++G(d,p)) of [(TPA)CoH]^+^ with thiophenol for the *S* = 1/2 (black) and the *S* = 3/2 spin state (red). *Calculated structures are depicted above or below the energy bar. Hydrogen atoms of the C–H bonds were removed for clarity.

Reaction 4 was observed only for the complexes with a free syn coordination site to the metal hydride bond. The metal complexes without a free coordination site, such as those supported by the TMC ligand, did not show reactivity toward the substrates. The requirement of the free coordination site indicates that in the gas phase, the substrate must first coordinate to the metal. Subsequently, the H_2_ elimination proceeds via proton migration within the reaction complex. In general, the reactivity decreases in the order Fe > Co > Ni, except for [(TPA)CoH]^+^ in the reaction with PhSH (Figure 6b, see Discussion below). For the complex [(Tpy)NiH]^+^ the reaction ends with the formation of a reaction complex without subsequent H_2_ elimination, likely due to their low hydride donor power (the lowest Δ*E*_CID_ among all studied complexes, see Figure S60). Hence, all experimental results (M–H stretching vibrations, BDEs, and reaction rates) suggest that the hydride donor ability of the metal hydrides with *N*-based ligands decreases in the order Fe > Co > Ni.

Finally, we have investigated the out-of-order reactivities of [(TPA)CoH]^+^ and [(TPA)FeH]^+^ with PhSH (Figure 7) by DFT. The [(TPA)MH]^+^ complexes have a trigonal bipyramidal geometry at the high spin-state configurations, whereas their low spin states are stabilized in the square pyramidal geometry (Figures S63–S64). [(TPA)NiH]^+^ and [(TPA)CoH]^+^ have a low spin ground state and, therefore, adopt the square pyramidal geometry (Figure 7). Conversely, [(TPA)FeH]^+^ has a high spin ground state; therefore, it adopts the trigonal bipyramidal geometry. The reaction of the [(TPA)MH]^+^ complexes with PhSH initially led to the formation of an adduct. The adduct formation with the PhSH reactant is more exothermic for the square pyramidal complex [(TPA)CoH]^+^ than for the trigonal bipyramidal complex [(TPA)FeH]^+^ because the former has an available free coordination site (see the structures and energies in Figure 7). Hence, the [(TPA)CoH]^+^ complexes react faster because their low spin configuration stabilizes the adduct with the thiophenol reactant in a distorted octahedral arrangement, leading to a smaller distortion of the complex geometry along the reaction coordinate.^18^ The same type of stabilization between thiophenol and the high spin complex [(TPA)FeH]^+^ requires a change in geometry, therefore representing an enthalpic and entropic energy penalty. Formic acid binds via the oxygen of the carbonyl, allowing a closer proximity between the proton and the hydride than in the case of the PhSH reactant (Figure S62). Hence, the reaction with formic acid requires fewer geometric changes along the reaction coordinate. In such a case, the barrier is driven by the complex hydricity. Accordingly, the reactivity trend of [(TPA)MH]^+^ with formic acid is as expected, that is, Ni < Co < Fe.

## Discussion

**Figure 8:**
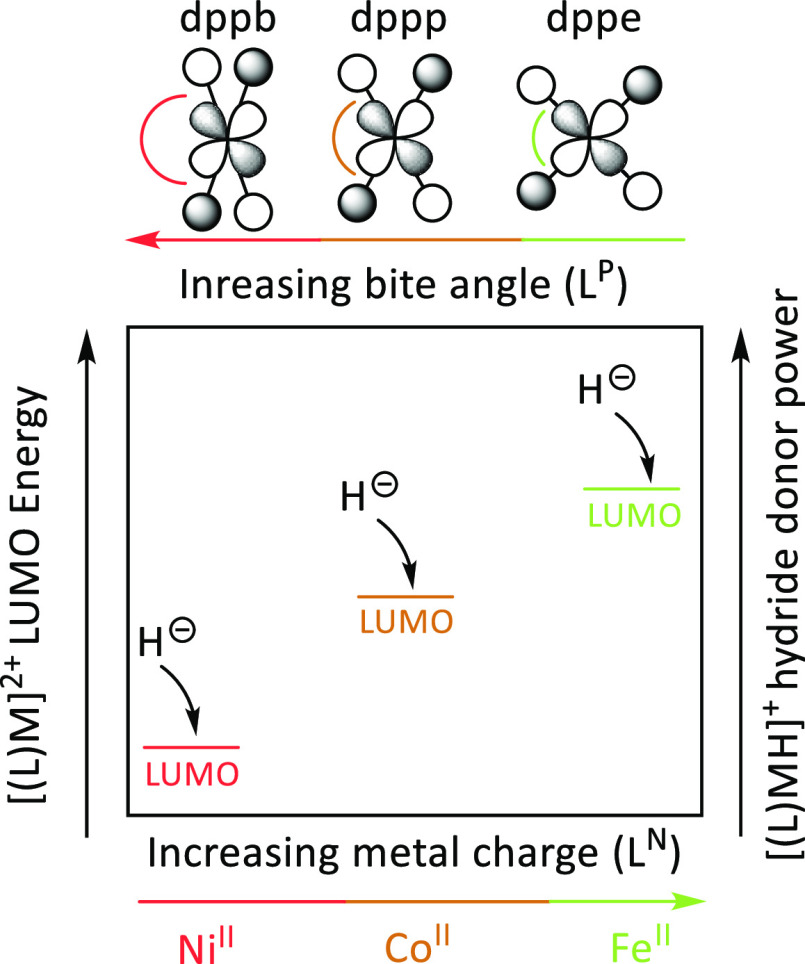
Qualitative orbital diagram of the LUMO energy of metal hydride acceptors [(L)M^ii^]^2+^ as a function of the charge on the metal center and of the phosphine bite angle.

We used several types of gas-phase experiments to estimate the hydricity trends of metal complexes. Hydricity is the free Gibbs energy for dissociating a hydride anion from the metal hydride. Hence, the hydricity is a thermodynamic parameter. The energy-resolved CID experiments are directly connected to the hydricity (Scheme 1 and eq 2), albeit with an unknown const(L) term error. The IR stretching frequencies of the M–H bonds reflect their bond strength. Both sets of data can be used to evaluate hydricity trends qualitatively. Contrarily, gas-phase collisional experiments reflect the hydride donor power in terms of kinetics (often called kinetic hydricity).^19^ As expected, there is a good correlation between the kinetics and the thermodynamics of hydride transfer reactions.^20^ The only exception among the studied series of metal hydride complexes is [(TPA)CoH]^+^. This complex exhibits larger reactivity with thiophenol because it has a low-spin configuration and a low-lying high-spin state.^21^ The access of both spin-state surfaces along the reaction coordinate drives the chemical transformation via a transition state with smaller distortion energies. While measurements of gas-phase photodissociation spectra of isolated ions and investigations of bimolecular reactivities are not always readily available experiments, investigations of the CID spectra of metal complexes are commonly available. Our results suggest that simple energy-resolved CID experiments can serve to estimate the hydricity trends of metal complexes. In determining the hydricity trends by measuring the activation energies for the dissociation of the [(L)M(BH_4_)]^+^ complexes, we assume that the activation energy depends on the ability of [(L)M]^2+^ to accept the hydride from the borohydride donor BH_4_^–^. The hydride donates its two electrons to the LUMO of the metal complex (Figure 8). The higher in energy is the LUMO of [(L)M]^2+^, the greater is the hydride donor ability of the corresponding metal hydride [(L)MH]^2+^. In the following, we discuss the trends given this assumption. The energy of the LUMO can be lowered by using a more electronegative metal. Accordingly, we have shown that the hydride donor ability decreases in the order Fe > Co > Ni complexes with the N-based ligands. However, such a trend does not hold for the complexes with P-based ligands. The explanation stems from the ligands’ electron-donating properties. The phosphine ligands are stronger σ-donors capable of counterbalancing the natural change of the metal when moving from Fe to Co and Ni. Natural bond orbital calculations show that the average charge on the metal center in the [(dppe)_2_MH]^+^ and [(dppp)_2_MH]^+^ complexes (M = Fe, Co, Ni) is 1.18 ± 0.02. On the other hand, the metal charge in the [(TPA)MH]^+^ complexes decreases by ca. 0.2 when going from Fe (1.32) to Co (1.09) and Ni (0.89). As a result, the Δ*E*_CID_ values of the metal complexes with N-based ligands are more affected by the change of the metal center than those with P-based ligands. Another parameter affecting the hydride donor ability is the bite angle of bidentate phosphines. The trend was observed for Co and Ni complexes with d^7^ and d^8^ electronic configurations, respectively.^22^ The LUMO of these complexes is described by the antibonding interaction between the d_*x*_^2^_–y_^2^ orbital of the metal and the phosphine orbitals (Figure 8). The LUMO orbital energy can be modulated by increasing or decreasing the overlap between the d_*x*_^2^_–y_^2^ and phosphine orbitals (Figure 8). The dppe ligand provides the best overlap with the d_*x*_^2^_–y_^2^ orbital, resulting in the larger Δ*E*_CID_ for [(dppe)CoH]^+^, followed by [(dppp)CoH]^+^ and [(dppb)CoH]^+^. A similar trend is observed for nickel complexes. The LUMO of the iron complexes with P-based ligands is not based on the metal d_*x*_^2^_–y_^2^ orbital; therefore, they do not exhibit a clear trend with the bite angle effect.

## Conclusions

Using gas-phase techniques, we present a new way to study the hydricity trends of transition metal hydrides. We have investigated iron, cobalt, and nickel complexes with three nitrogen- and three phosphorus-based ligands. These complexes formed metal hydrides [LM(H)]^+^ by the elimination of BH_3_ from their metal borohydride precursors [LM(BH_4_)]^+^. The appearance energy of this fragmentation (Δ*E*_CID_) correlates well with the gas-phase hydricities. We have characterized the [LM(H)]^+^ complexes by their vibrational and electronic spectra, allowing us to assign the generated complexes’ spin states. The M–H stretching frequency (*ν*(M–H)) correlated well with the Δ*E*_CID_ appearance energies for forming the metal hydride complexes. Finally, we investigated the hydride donor abilities of the complexes in the bimolecular reactions of the complexes with PhSH and HCOOH, which also correlated well with the trend predicted based on Δ*E*_CID_ and *ν*(M–H). The results rationalize the effects of the metal center, ligand electronics, and ligand geometries on the hydricity of the respective metal hydrides.

## Experimental Section

### Generation of the [(L)M(BH_4_)]^+^ and [(L)M(H)]^+^ Complexes

The ligands (tris(2-pyridylmethyl)amine (TPA), tetramethylcyclam (TMC), terpyridine (Tpy), diphosphinephenylethene (dppe), diphosphinephenylpropane (dppp), diphosphinephenylbutane (dppb), and diphosphinemethylethane (dpme)) and metal triflate salts were purchased from Sigma-Aldrich and used without further purification. Stock solutions (1.0 mM) of the metal complexes were prepared by mixing the ligands with metal triflate salts [M(TfO)_2_] (M = Fe, Co, Ni) in acetonitrile in a 1:1 ratio. To prevent ligand oxidation, the preparation and storage of metal complexes with phosphine-based ligands were performed in a glovebox. Finally, the borohydride adducts [(L)M(BH_4_)]^+^ were prepared in a flow reactor directly connected to the electrospray source of the mass spectrometer. The flow reactor was fed with a solution of the metal(II)-complex [(L)M(OTf)_2_] (0.1 mmol L^–1^) and with a solution of sodium borohydride (2.5 mmol L^–1^). Reaction times were kept in the range of ∼5 s to avoid the decomposition of the borohydride adducts. The metal-hydride complexes were generated by collision-induced dissociation of the borohydride adducts in the source (induced by high voltage differences between the capillary and tube lens during the transfer and high capillary temperature of ∼200 °C).

### Bond Dissociation Energies

The experiments were performed with the LCQ Deca XP (Finnigan) ion trap mass spectrometer.^23^ The metal borohydride adducts formed in solution were transferred to the gas phase by electrospray ionization (ESI) at mild conditions (low voltage difference during transfer, capillary temperature ∼200 °C). The ^12^C/^11^B isotope was mass-selected, transferred to the quadrupole ion trap, and studied by CID. The relative fragmentation cross-section of the BH_3_ elimination in dependence on the collisional energy can be used to determine the bond dissociation energy (BDE)^24^. The normalized collision energy in the LCQ instruments can be converted to the center-of-mass collision energies by calibration with a series of thermometer ions (benzylpyridinium ions) with known BDEs.^24a^ The BDE corresponds to the onset of the linear extrapolation of the sigmoid fit of the fragmentation cross-section curve. The prerequisite of these experiments is that the collision energy in the given ion trap scales with the *m*/*z* ratio of the mass-selected ions (this condition is fulfilled for the LCQ ion traps^25^).

### Infrared Photodissociation Spectra

The helium or neon tagging IRPD spectra were obtained with the ISORI instrument described in detail previously.^26^ Generation of the hydride complexes was accomplished using the procedure described above. The main isotope was mass-selected by the first quadrupole and transferred via a quadrupole bender and an octopole to a cryogenic (∼3 K) ion trap. The ions were trapped and thermalized using pulsed helium or a helium/neon mixture (10:1), and weakly bound complexes with He or Ne formed. The complexes were irradiated with a tunable IR beam. Absorption of an IR photon causes dissociation of the helium/neon complexes. Hence, the depletion of helium/neon complexes w.r.t. IR wavenumber provides the IRPD spectrum. The metal-hydride vibration was assigned by the isotopic shifts in the IR spectra upon labeling a metal-hydride to a metal-deuteride (using NaBD_4_ for its generation).

### Ion–Molecule Reactivity Studies

A triple quadrupole mass spectrometer, TSQ 7000, was used to perform the gas-phase reactivity studies of 6 metal-hydride complexes. The metal hydride ions were generated identically according to the procedure described above. The main isotopologue was mass-selected by the first quadrupole, and the nominal collision energy was set to 0 eV, as determined by retarding potential analysis (Figure S51). The kinetic energy distribution of ions (fwhm = ∼ 0.8 eV) was tuned to be identical (max 10% deviation) for all measurements to keep the same conditions within the experiments. The metal-hydride complexes were guided to the octopole collision cell at 313 K, filled with gaseous thiophenol or formic acid (<0.35 mTorr). The relative reaction rates were extracted from the dependence of the relative cross-section of the reaction product on the pressure. The slope of the linear fit of the cross-section pressure dependence gave the relative reaction rate constants.^27^

### Density Functional Theory Calculations

The calculations were performed with the B3LYP^28^ functional and D3 dispersion correction,^29^ and the triple-ζ basis set 6-311++G**^30^ as implemented in the Gaussian 16 program.^31^ The Hessian calculations confirmed all minima and provided harmonic IR spectra, to which we applied a scaling factor of 0.98. We always screened multiple spin isomers, geometrical isomers, and conformations and reported the most stable structures.

## Supporting Information Available

Additional experimental details and methods, all experimental and calculated spectra, fragmentation breakdown diagrams, gas-phase reactivities, and theoretical results (PDF)
Calculated structures and zero-point energies (XYZ)

## Supplementary Material

ja3c08925_si_001.pdf

ja3c08925_si_002.xyz
