# Supplementary material for: Properties of Metal Hydrides of the Iron Triad
Source: J Am Chem Soc. 2023 Dec 7;145(50):27555–62. doi: 10.1021/jacs.3c08925 (PMC10740003; doi:10.1021/jacs.3c08925)
Supplement: Supplementary file 1 — ja3c08925_si_001.pdf [file ja3c08925_si_001.pdf]

Supporting Information for Properties of metal hydrides of the iron triad.

Arie J. H. Multem, Guilherme L. Tripodi, Jana Roithová*

Department of Spectroscopy and Catalysis, Institute for Molecules and Materials, Radboud University Nijmegen, Heyendaalseweg 135, 6525 AJ Nijmegen, The Netherlands

Contents

IRPD spectra 2
Experimental details 2
IR Spectra of all measured ions 4
Results 10
Bond dissociation energies 12
Experimental details 12
Spectra and breakdown diagrams: N-based ligands 13
Spectra and breakdown diagrams: P-based ligands 19
Results 25
Gas phase reactivity studies 26
Experimental details 26
Gas phase reactivity mass spectra 26
Results 30
Density Functional Theory studies 31
Potential energy surfaces and structures 31
Results 32

IRPD spectra

Experimental details

A home-build IRPD ESI-MS , the ISORI instrument, was used to acquire the IRPD spectra (see the experimental part in the main document). The exact pulse sequence is given below in Figure S1. The number of the cycles and the length of the pulses could vary between ions to optimize the helium- or neon-tagged ions' signal. When neon-tagging was used, a 10% mixture of neon in helium was used.

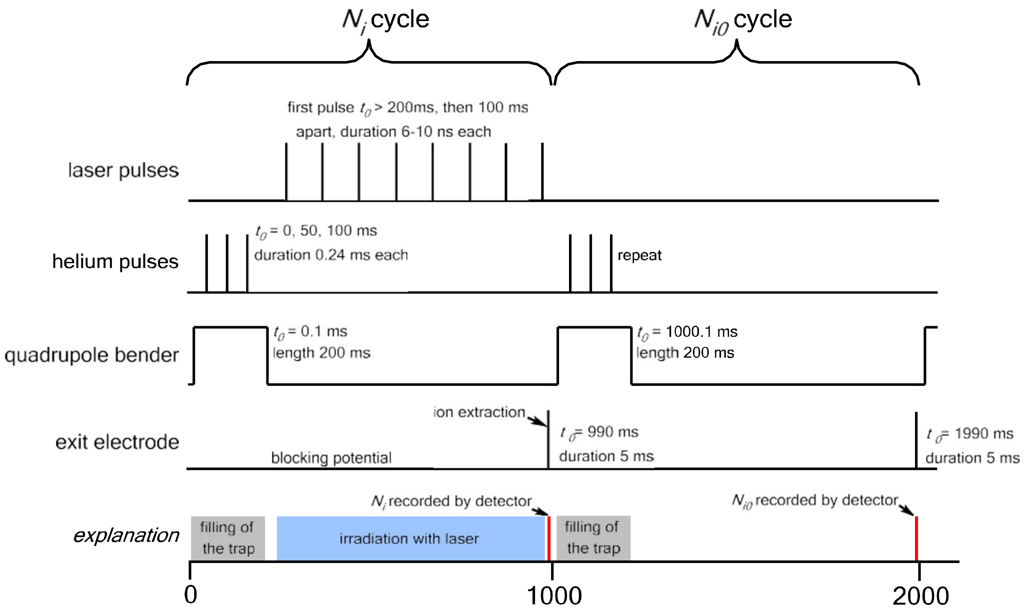

Figure S1. Pulse sequence for the acquisition of IR spectra by the ISORI instrument.

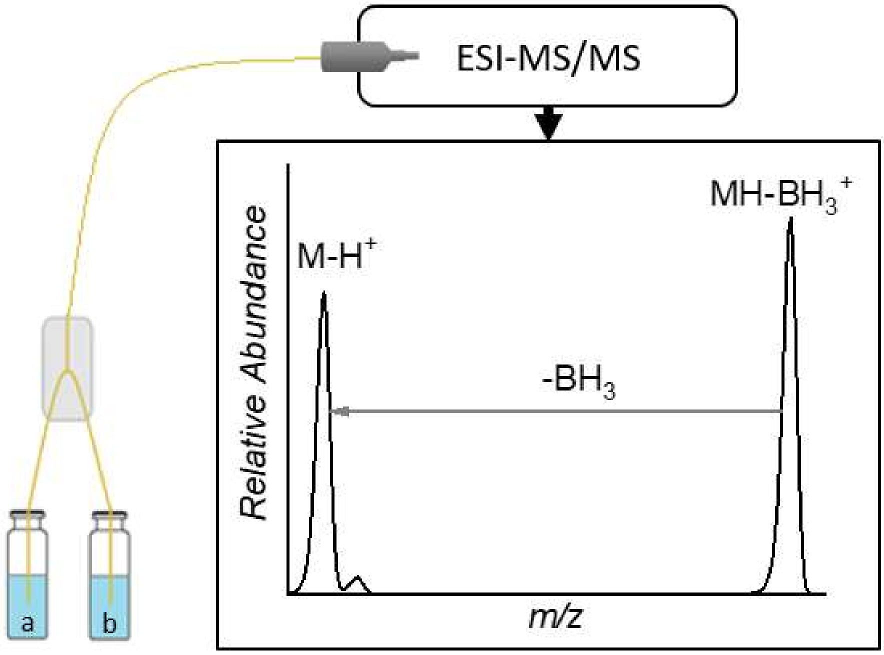

Figure S2. The schematic setup used to generate the metal borohydride and metal hydride complexes. The borohydride adducts were prepared by mixing the metal precursor $[LM]^{2+}$ (a) with a $NaBH_4$ solution (b) in MeCN with a homemade flow reactor. The metal hydride complex was generated by the fragmentation of the BH_3 fragment from the metal borohydride complex.

Preparation of solutions for ESI:

$NaBH_4$/BD_4 : 1.9 mg of $NaBH_4$ (5 mmol) was dissolved in 10 mL acetonitrile by sonication and diluted to 2.5 mM.

$[(TPA)FeH]^+$: A 0.1 mM solution of FeTPA was prepared by mixing 3.5 mg of Fe(II)OTf_2 (1 mmol) with 2.9 mg tris(2-pyridylmethyl)amine (TPA)(1 mmol) in 10 mL acetonitrile. The solution was sonicated for ~5 minutes and diluted to 0.1 mM with acetonitrile.

$[(TPA)CoH]^+$: A 0.1 mM solution of CoTPA was prepared by dissolving 13.2 mg $[Co(TPA)(Cl)_2]$ (1 mmol) in 10 mL acetonitrile by sonication and diluting to 0.1 mM with acetonitrile.

All other metal complexes were prepared analogously to the method described for $[(TPA)FeH]^+$. The metal complex solution and $NaBH_4$ solution were mixed together with a homemade mixing T-piece before spraying. The flow rate could be controlled manually by overpressure N_2 gas. A schematic of the setup is depicted in Figure S2.

TPA's: Spraying conditions varied per ion and were optimized before every experiment; a typical experiment employed the following spraying conditions: spray voltage: 3.5 kV, capillary temperature: 250 °C, capillary voltage: 60 V, tube lens: 110 V, N_2 sheath gas: 30 psi. A high capillary voltage and tube lens difference was needed to break the boron hydride precursor ion and generate maximum signal intensity for the metal hydride ion.

TMC's: Spraying conditions varied per ion and were optimized before every experiment; a typical experiment employed the following spraying conditions: spray voltage: 3.5 kV, capillary temperature: 250 °C, capillary voltage: 50 V, tube lens: 150 V, N_2 sheath gas: 30 psi. A high capillary voltage and tube lens difference was needed to break the boron hydride precursor ion and generate maximum signal intensity for the metal hydride ion.

Tpy's: Spraying conditions varied per ion and were optimized before every experiment; a typical experiment employed the following spraying conditions: spray voltage: 3.5 kV, capillary temperature: 250 °C, capillary voltage: 25 V, tube lens: 110 V, N_2 sheath gas: 30 psi. A high capillary voltage and tube lens difference was needed to break the boron hydride precursor ion and generate maximum signal intensity for the metal hydride ion.

IR Spectra of all measured ions

[(TPA)FeH]:

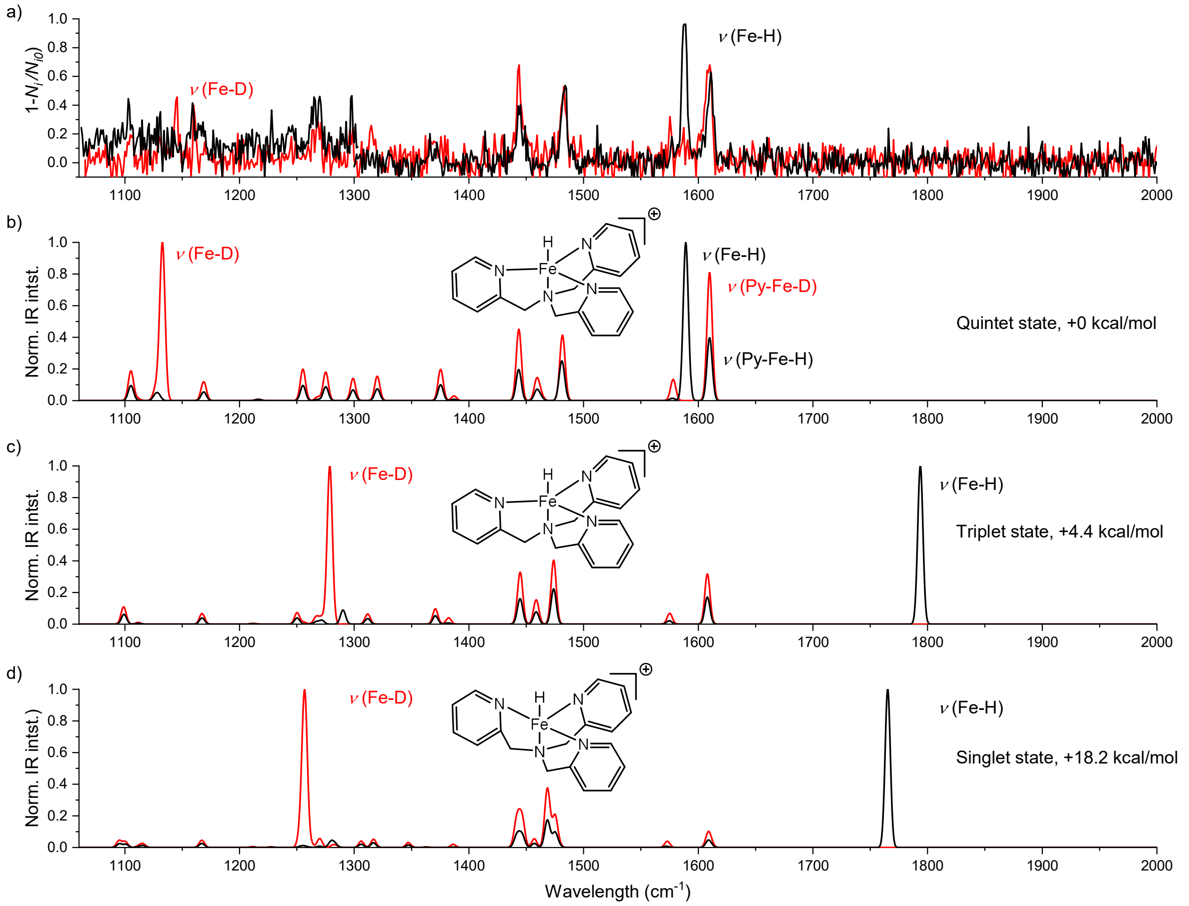

Figure S3. Comparison of the experimental spectrum (a) of $[(TPA)FeH]^+$ and calculated spectra (b-d) of the different spin states at the B3LYP-D3 level of theory and 6-311++G** basis set. Energies relative to the lowest-lying spin state are given on the right in kcal/mol. The depicted conformer was calculated to be the lowest-lying one.

[(TPA)CoH]$^+$:

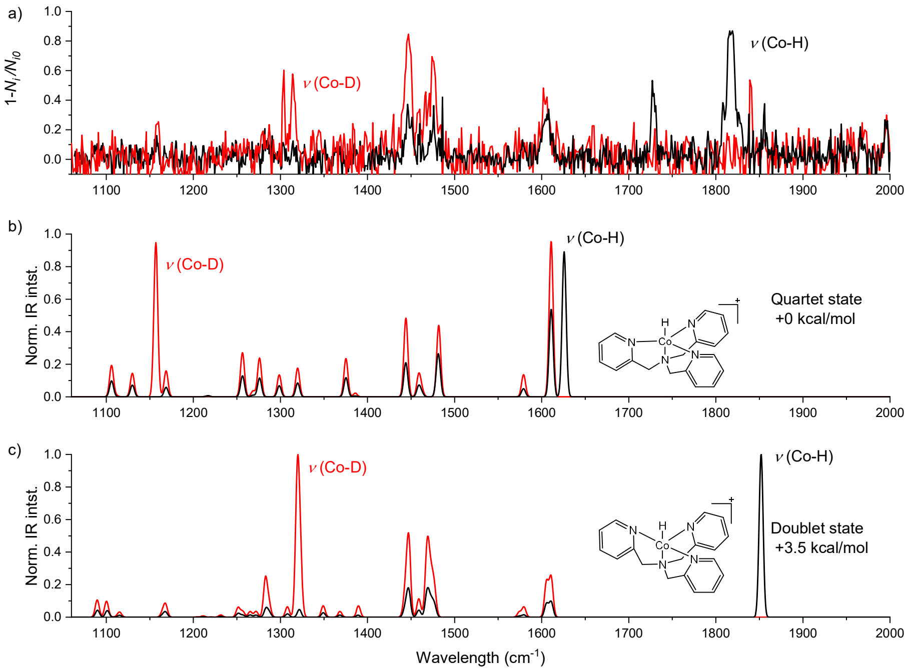

Figure S4. Comparison of the experimental spectrum (a) of [(TPA)CoH]$^+$ and calculated spectra (b-c) of the different spin states at the B3LYP-D3 level of theory and 6-311++G** basis set. Energies relative to the lowest-lying spin state are given on the right in kcal/mol. The depicted conformer was calculated to be the lowest-lying one.

[(TPA)Ni(H)]$^+$:

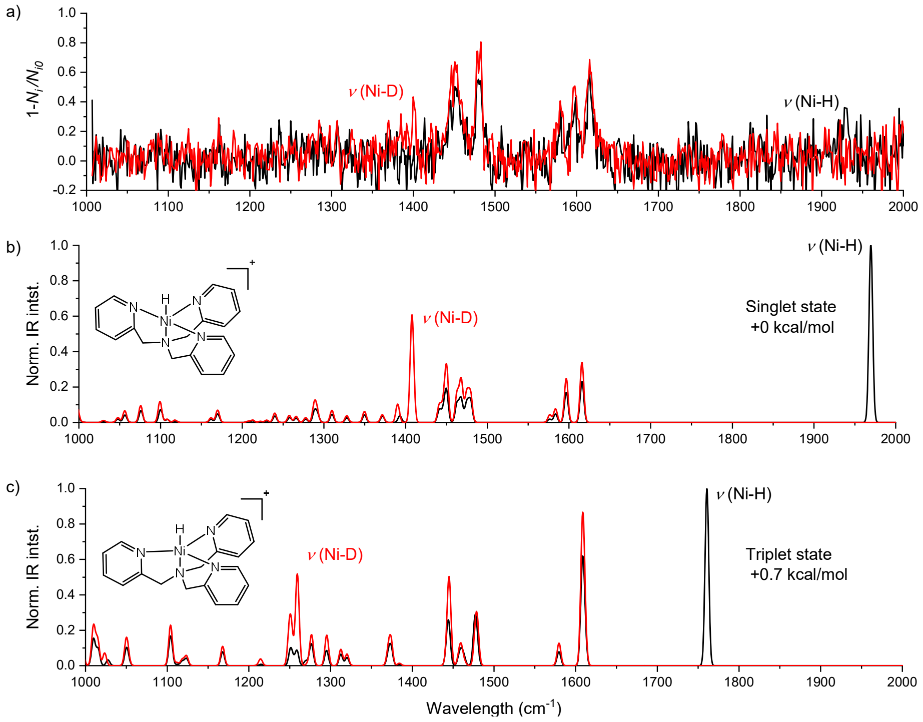

Figure S5. Comparison of the experimental spectrum (a) of [(TPA)NiH]$^+$ and calculated spectra (b-c) of the different spin states at the B3LYP-D3 level of theory and 6-311++G** basis set. Energies relative to the lowest-lying spin state are given on the right in kcal/mol. The depicted conformer was calculated to be the lowest-lying one.

[(TMC)Fe(H)]+ :

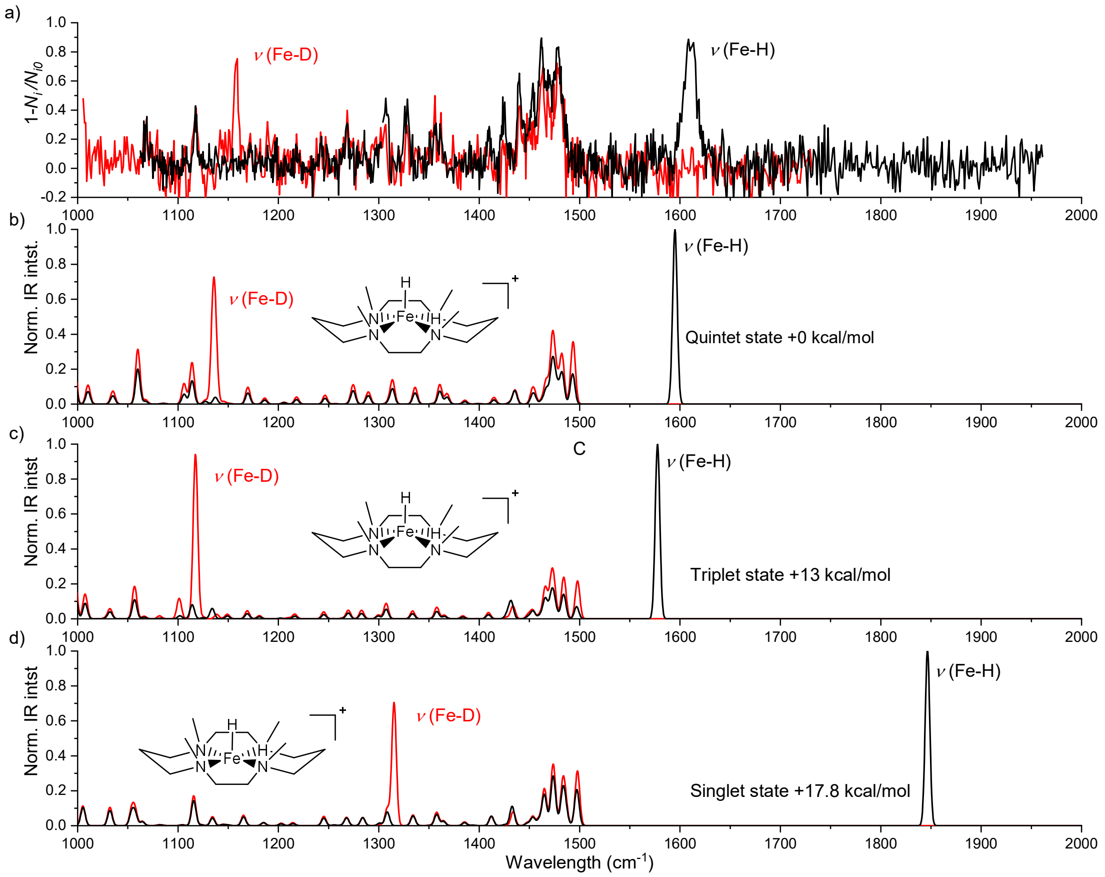

Figure S6. Comparison of the experimental spectrum (a) of $[(TMC)FeH]^+$ and calculated spectra (b-c) of the different spin states at the B3LYP-D3 level of theory and 6-311++G** basis set. Energies relative to the lowest-lying spin state are given on the right in kcal/mol. The depicted conformer was calculated to be the lowest-lying one.

$[(TMC)CoH]^+$:

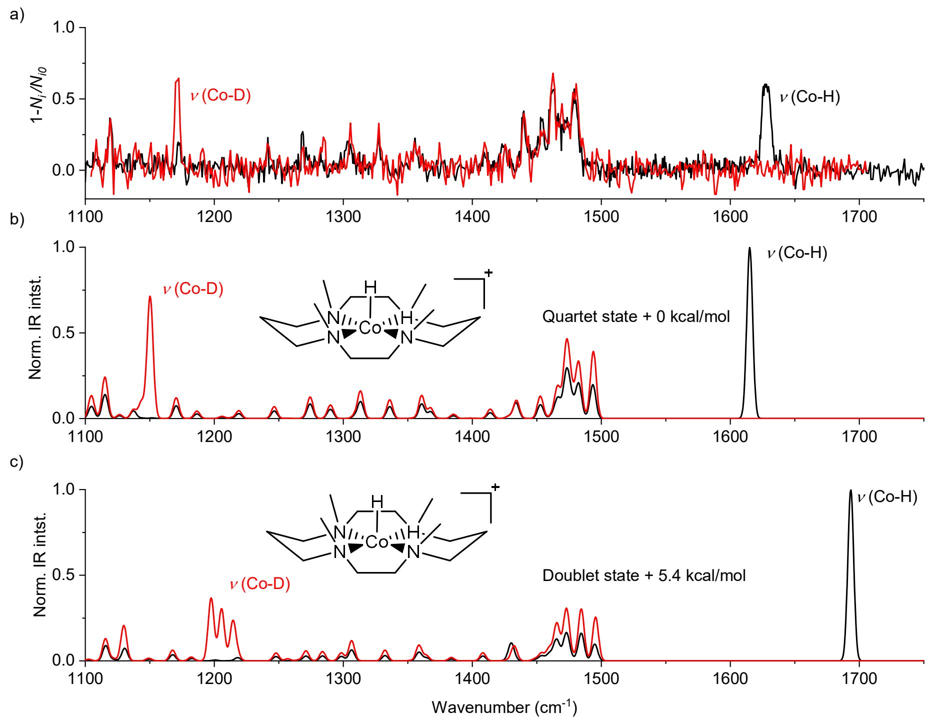

Figure S7. Comparison of the experimental spectrum (a) of $[(TMC)CoH]^+$ and calculated spectra (b-c) of the different spin states at the B3LYP-D3 level of theory and 6-311++G** basis set. Energies relative to the lowest lying spin state are given in kcal/mol. The depicted conformer was calculated to be the lowest-lying one.

$[(TMC)NiH]^+$:

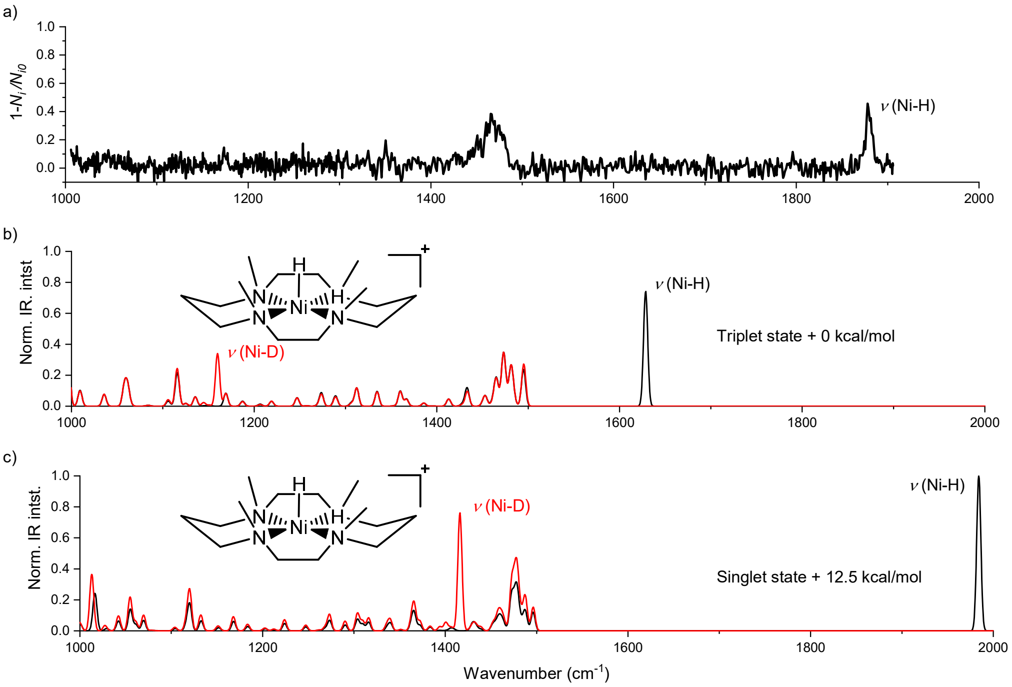

Figure S8. Comparison of the experimental spectrum (a) of $[(TMC)NiH]^+$ and calculated spectra (b-c) of the different spin states at the B3LYP-D3 level of theory and 6-311++G** basis set. Energies, relative to the lowest lying spin state, are given in kcal/mol. The depicted conformer was calculated to be the lowest-lying one.

[(Tpy)FeH]⁺:

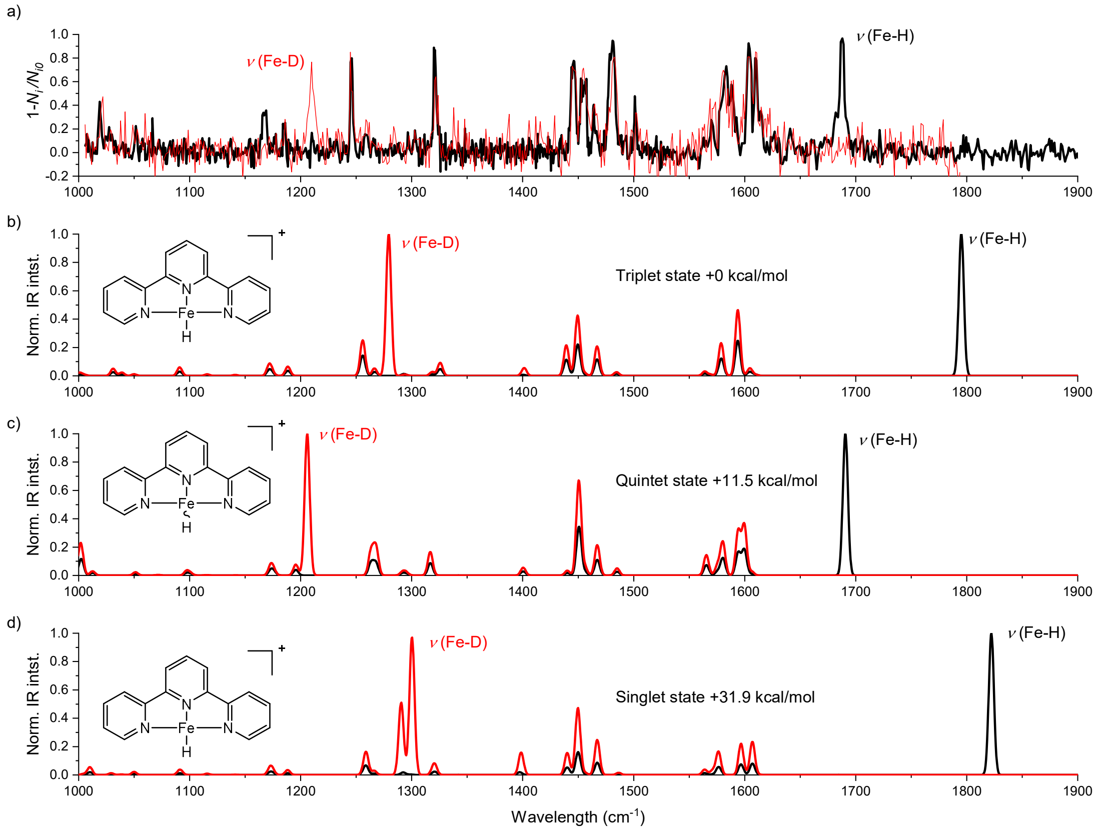

Figure S9. Comparison of the experimental spectrum (a) of $[(Tpy)FeH]^+$ and calculated spectra (b-d) of the different spin states at the B3LYP-D3 level of theory and 6-311++G** basis set. Energies are relative to the lowest-lying spin state and are given in kcal/mol. The depicted conformer was calculated to be the lowest-lying one.

[(Tpy)CoH]⁺:

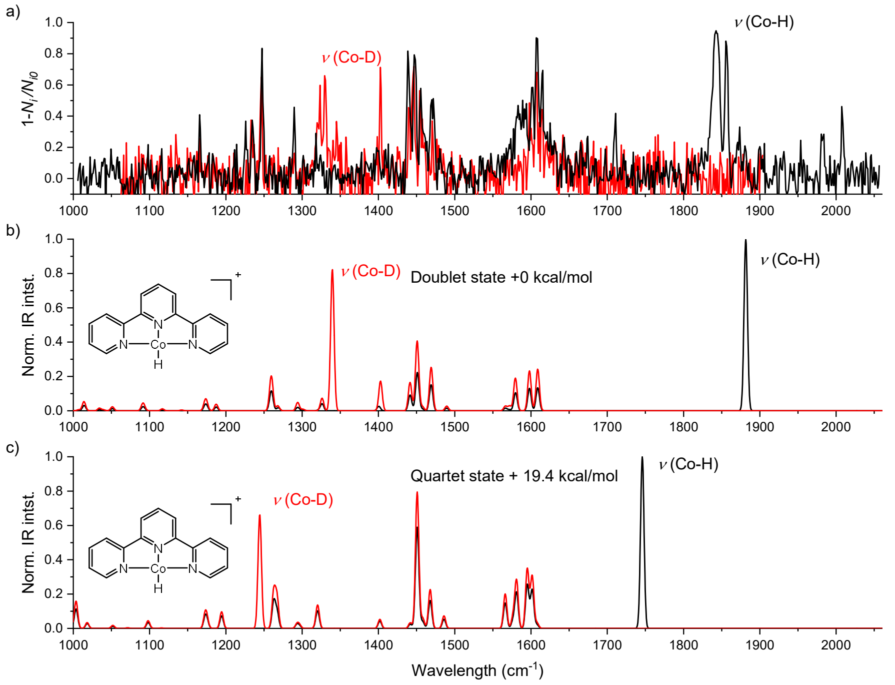

Figure S10. Comparison of the experimental spectrum (a) of $[(Tpy)CoH]^+$ and calculated spectra (b-c) of the different spin states at the B3LYP-D3 level of theory and 6-311++G** basis set. Energies relative to the lowest-lying spin state are given in kcal/mol. The depicted conformer was calculated to be the lowest-lying one.

[(Tpy)NiH]⁺:

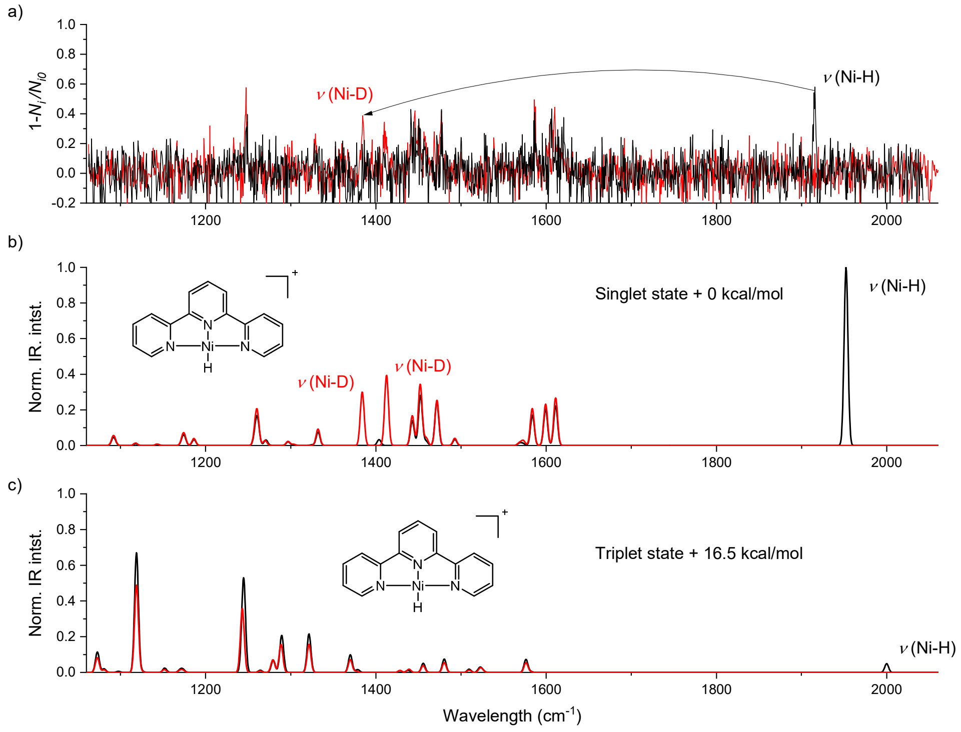

Figure S11. Comparison of the experimental spectrum (a) of $[(Tpy)NiH]^+$ and calculated spectra (b-c) of the different spin states at the B3LYP-D3 level of theory and 6-311++G** basis set. Energies relative to the lowest-lying spin state are given in kcal/mol. The depicted conformer was calculated to be the lowest-lying one.

Results

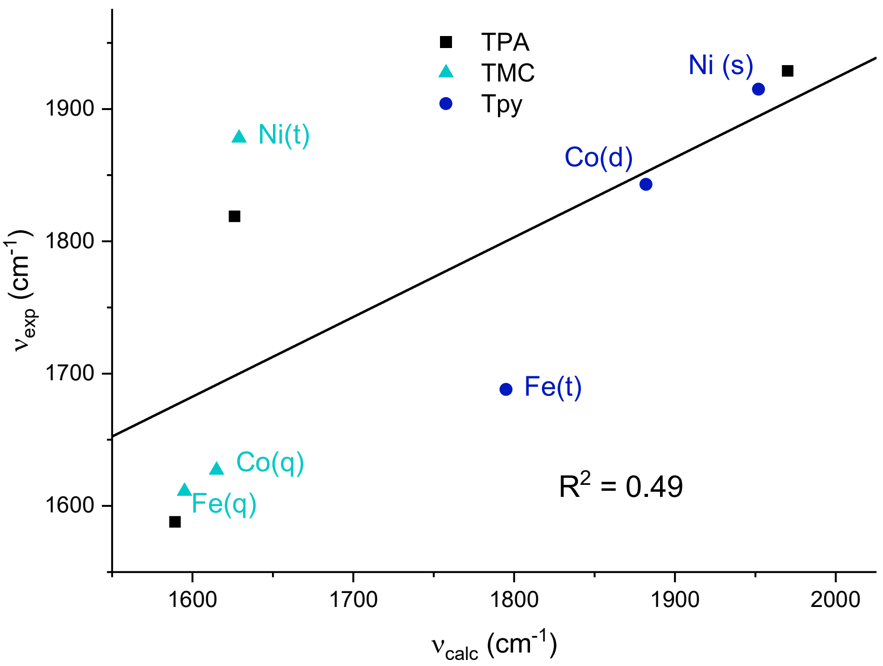

Figure S12. Linear regression of the experimentally obtained ν(M-H) (table 1) versus the calculated ν(M-H) of $[(L)MH]^+$ with L = TPA, TMC and Tpy and M = Fe, Co, Ni. The calculated ν(M-H) of the lowest-lying spin state is shown. Electronic configuration is given in parenthesis behind the metal. Calculated at B3LYP/6-311++G**.

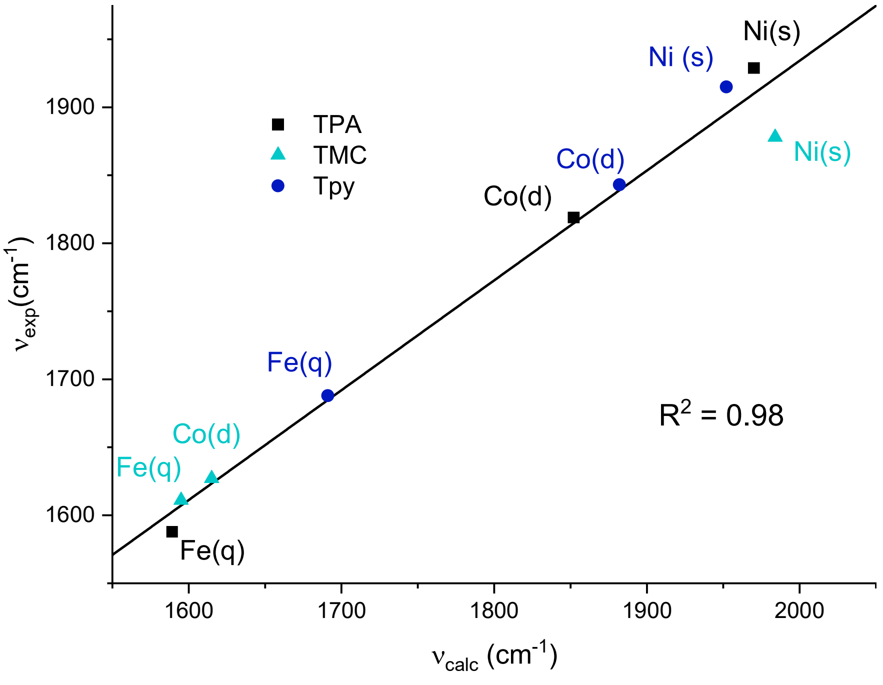

Figure S13. Linear regression of the experimentally obtained ν(M-H) (table 1) versus the calculated ν(M-H) of $[(L)MH]^+$ with L = TPA, TMC and Tpy and M = Fe, Co, Ni. The calculated ν(M-H) is shown of the spin state with the best spectral match with the experimental spectrum. Electronic configuration given in parenthesis behind metal. Calculated at B3LYP/6-311++G**.

VIS spectrum of [(TPA)CoH]$^+$

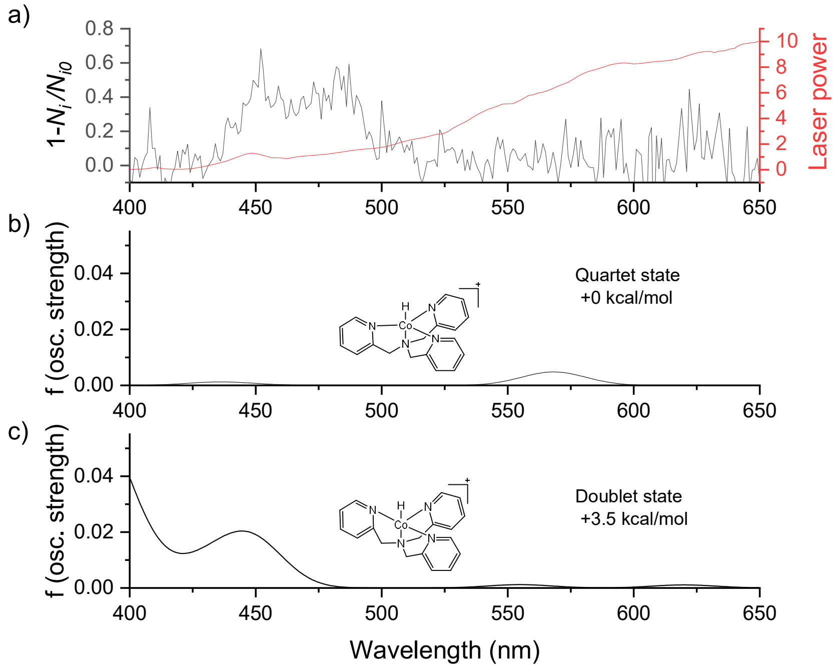

Figure S14. Comparison of the experimental spectrum (a) of [(TPA)CoH]$^+$ and calculated spectra (b-c) of the different spin states at B3LYP-D3/6-311++G**. Energies relative to the lowest-lying spin state are given in kcal/mol. The depicted conformer was calculated to be the lowest-lying one.

Table S1. Overview of experimentally obtained metal hydride vibrations and the calculated metal hydride vibration of the assigned spin state based on the spectral match.

**METAL HYDRIDE COMPLEX**	experimental ν(M-H) (cm^{-1})	Calculated ν(M-H) (cm^{-1}) (B3LYP/6-311++G*)
[(TPA)Fe(H)]$^+$	1588	1589 (S=2)
[(TPA)Co(H)]$^+$	1819	1852 (S=1/2)
[(TPA)Ni(H)]$^+$	1929	1970 (S=0)
[(TMC)Fe(H)]$^+$	1611	1595 (S=2)
[(TMC)Co(H)]$^+$	1627	1615 (S=3/2)
[(TMC)Ni(H)]$^+$	1878	1984 (S=0)
[(TMC)Fe(H)]$^+$	1688	1691 (S=2)
[(Tpy)Co(H)]$^+$	1843	1882 (S=1/2)
[(Tpy)Ni(H)]$^+$	1915	1952 (S=0)

Bond dissociation energies

Experimental details

N-based ligands: Metal complexes were prepared analogously to the method described in the first section. The metal complex and BH_4 solution were mixed with a homemade mixing piece before spraying. The flow rate could be controlled manually by overpressure N_2 gas. A schematic of the setup is depicted in Figure S2.

P-based ligands:

Preparation of solutions for ESI:

$NaBH_4$: 1.9 mg of $NaBH_4$ (5 mmol) was dissolved in 10 mL dry acetonitrile and diluted to 2.5 mM before use.

$[(dppx)_2M(BH_4)]^+$, x = e, p, b, M = Fe, Co, Ni: All solutions containing the phosphine ligands were prepared in the glovebox to prevent ligand oxidation. A 0.1 mM solution of Mdppx was prepared by mixing 1 mmol of M(II)OTf_2 with 1 mmol of diphosphine ligand in 10 mL dry acetonitrile. The solution was left overnight, diluted to 0.1 mM with dry acetonitrile, and immediately transferred to the mass spectrometer before use.

$[(dmpe)_2Fe(BH_4)]^+$: A 0.1 mM solution of Fedmpe was prepared by mixing 3.5 mg of Fe(II)OTf_2 (1 mmol) with 1.7 µL of 1,2-Bis(dimethylphosphino)ethane (1.5 mg, 1 mmol) in 10 mL acetonitrile. The solution was sonicated for ~5 min and diluted to 0.1 mM with acetonitrile.

The metal complex solution and $NaBH_4$ solution were mixed with a homemade mixing piece before spraying. The flow rate could be controlled manually by overpressure N_2 gas. A schematic of the setup is depicted in Figure S2.

N-based ligands: Spraying conditions varied per ion and were optimized before every experiment; a typical experiment employed the following spraying conditions: spray voltage: 5.0 kV, capillary temperature: 200 °C, capillary voltage: 5 V, tube lens: 10 V, N_2 sheath gas: 20 psi. The capillary voltage- and tube lens difference was kept small to prevent breaking the boron hydride ion.

P-based ligands: Spraying conditions varied per ion and were optimized before every experiment; the experiments employed the following spraying conditions:

$[(dppx)_2M(BH_4)]^+$, x = e, p, b, M = Fe, Co: Spray voltage: 5.0 kV, capillary temperature: 220 °C, capillary voltage: 5 V, tube lens: 10 V, N_2 sheath gas: 20 psi. The capillary voltage and tube lens difference was kept small to prevent breaking the boron hydride ion.

$[(dppp)_2Ni(BH_4)]^+$: Spray voltage: 5.0 kV, capillary temperature: 170 °C, capillary voltage: 5 V, tube lens: 10 V, N_2 sheath gas: 20 psi. The capillary voltage and tube lens difference was kept small to prevent breaking the boron hydride ion.

$[(dppb)_2Ni(BH_4)]^+$: Spray voltage: 5.0 kV, capillary temperature: 200 °C, capillary voltage: 5 V, tube lens: 10 V, N_2 sheath gas: 20 psi. The capillary voltage and tube lens difference was kept small to prevent breaking the boron hydride ion.

$[(dmpe)_2Fe(BH_4)]^+$: Spray voltage: 5.0 kV, capillary temperature: 200 °C, capillary voltage: 5 V, tube lens: 10 V, N_2 sheath gas: 20 psi. The capillary voltage and tube lens difference was kept small to prevent breaking the boron hydride ion.

Spectra and breakdown diagrams: N-based ligands

$[(TPA)Fe(BH_4)]^+$:

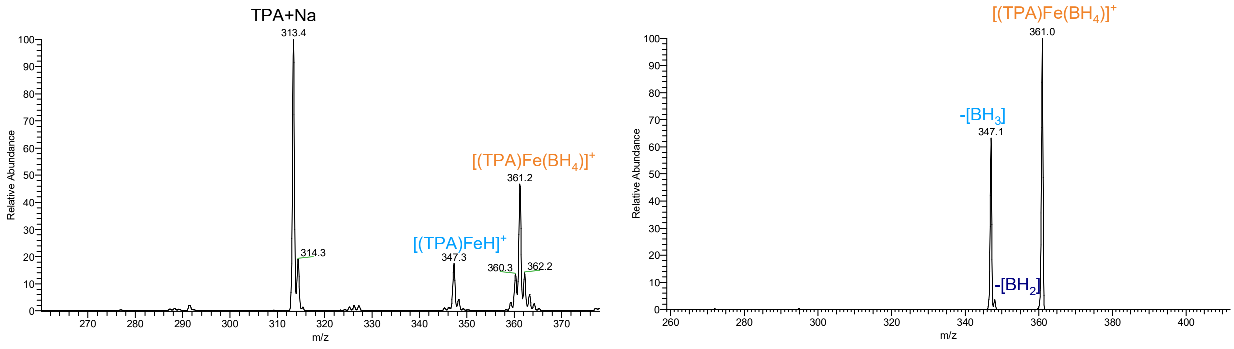

Figure S15. ESI-MS spectrum of a mixture of iron complex and sodium borohydride (left). CID spectrum of mass-selected $[(TPA)Fe(BH_4)]^+$ (right).

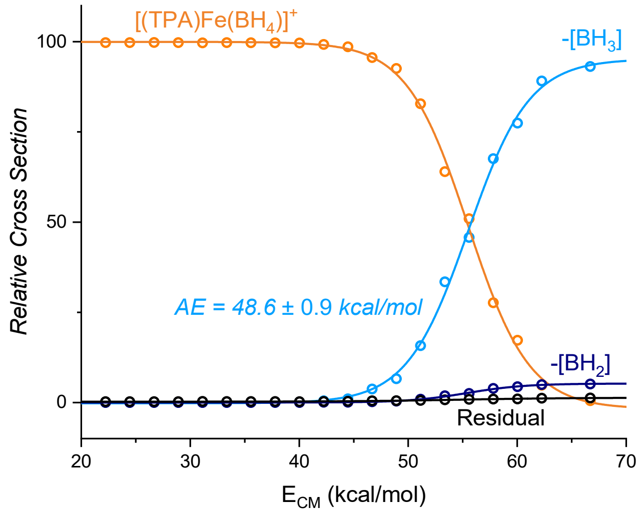

Figure S16. Breakdown diagram of $[(TPA)Fe(BH_4)]^+$, the dots are experimental data, the lines are fitted sigmoid functions. Linear extrapolation gives us the appearance energy (AE) or bond dissociation energy (BDE) of the fragmentation.

$[(TPA)Co(BH_4)]^+$:

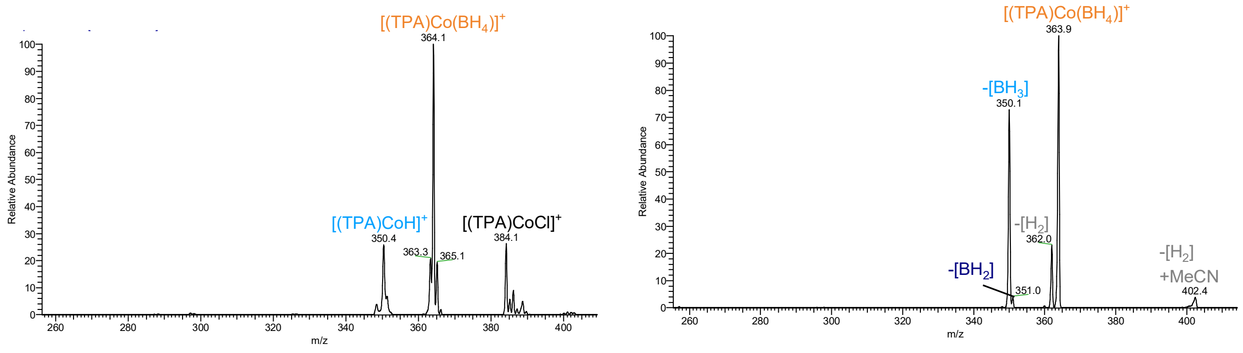

Figure S17. ESI-MS spectrum of a mixture of cobalt complex and sodium borohydride (left). CID spectrum of mass-selected $[(TPA)Co(BH_4)]^+$ (right)

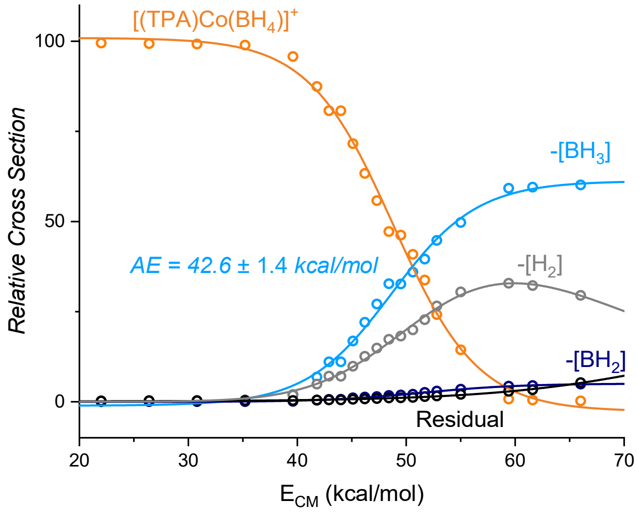

Figure S18. Breakdown diagram of [(TPA)Co(BH₄)]⁺, the dots are experimental data, the lines are fitted sigmoid functions. Linear extrapolation gives us the appearance energy (AE) or bond dissociation energy (BDE) of the fragmentation.

[(TPA)Ni(BH₄)]⁺:

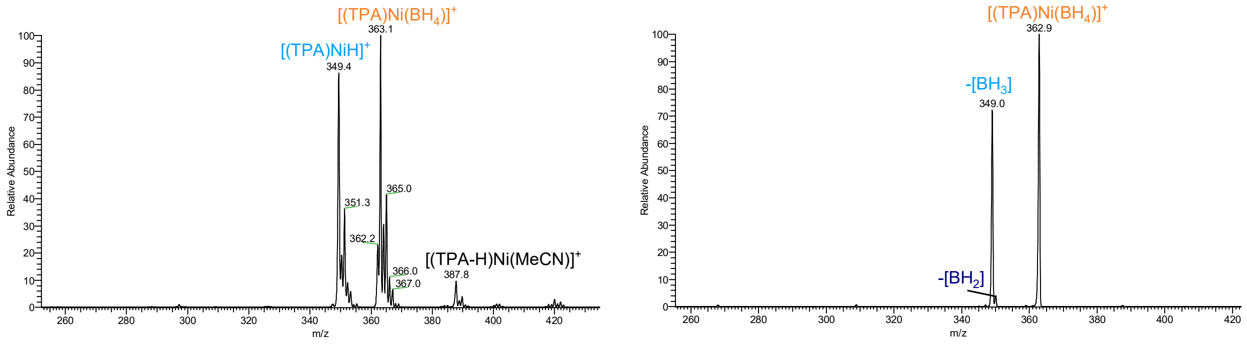

Figure S19. ESI-MS spectrum of a mixture of nickel complex and sodium borohydride (left). CID spectrum of mass-selected [(TPA)Ni(BH₄)]⁺ (right).

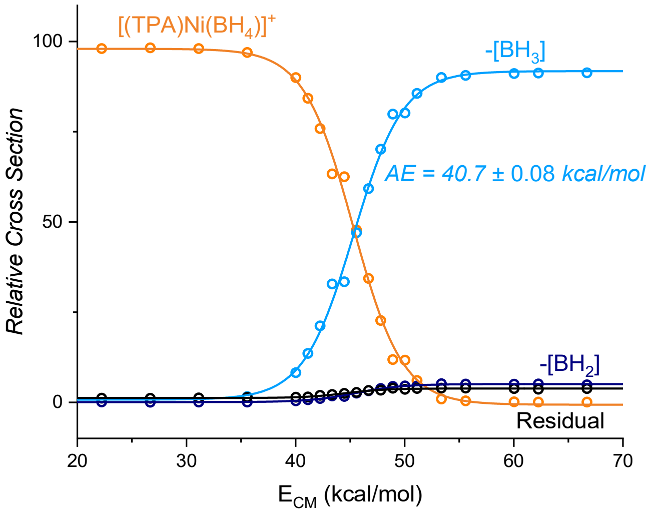

Figure S20. Breakdown diagram of [(TPA)Ni(BH₄)]⁺, the dots are experimental data, the lines are fitted sigmoid functions. Linear extrapolation gives us the appearance energy (AE) or bond dissociation energy (BDE) of the fragmentation.

[(TMC)Fe(BH_4)]$^+$:

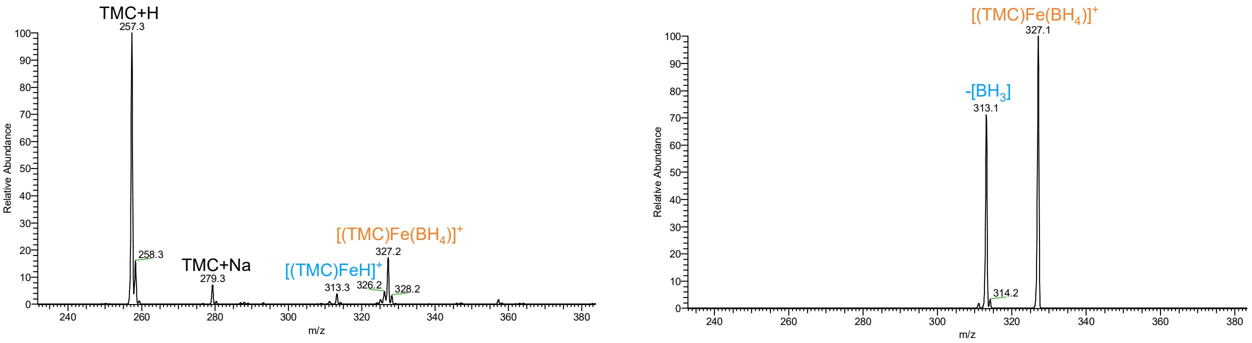

Figure S21. ESI-MS spectrum of a mixture of iron complex and sodium borohydride (left). CID spectrum of mass-selected [(TMC)Fe(BH_4)]$^+$ (right).

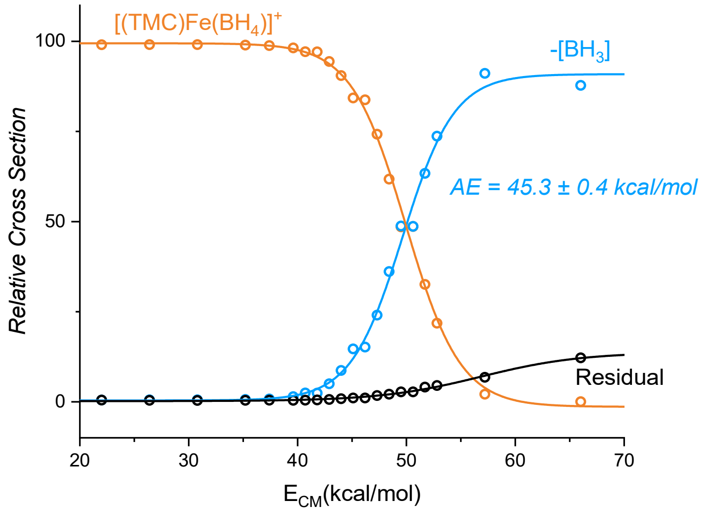

Figure S22. Breakdown diagram of [(TMC)Fe(BH_4)]$^+$, the dots are experimental data, the lines are fitted sigmoid functions. Linear extrapolation gives us the appearance energy (AE) or bond dissociation energy (BDE) of the fragmentation.

[(TMC)Co(BH_4)]$^+$:

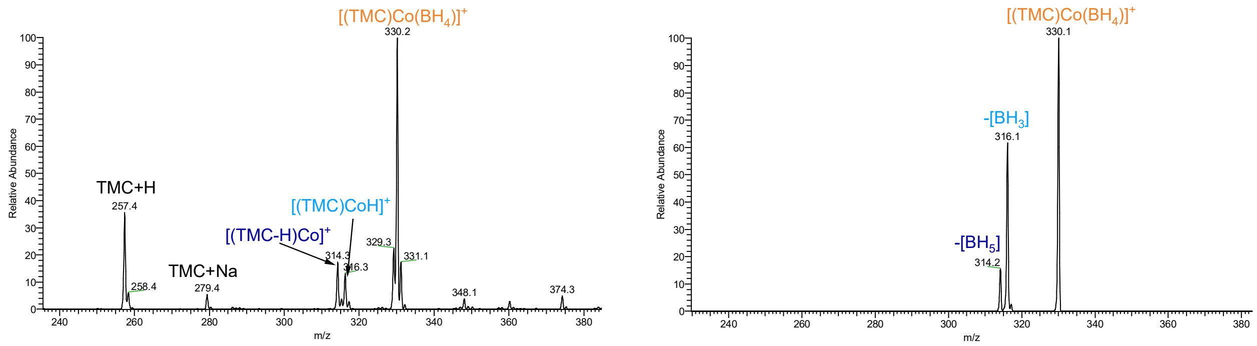

Figure S23. ESI-MS spectrum of a mixture of cobalt complex and sodium borohydride (left). CID spectrum of mass-selected [(TMC)Co(BH_4)]$^+$ (right).

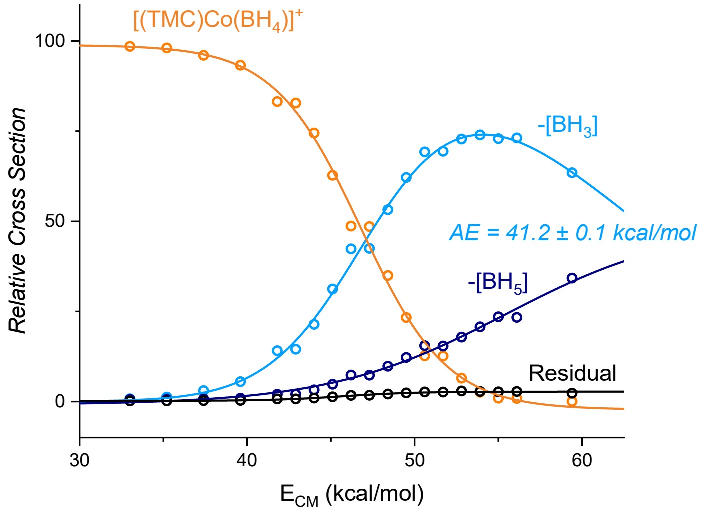

Figure S24. Breakdown diagram of [(TMC)Co(BH$_4$)]$^+$, the dots are experimental data, the lines are fitted sigmoid functions. Linear extrapolation gives us the appearance energy (AE) or bond dissociation energy (BDE) of the fragmentation.

[(TMC)Ni(BH$_4$)]$^+$:

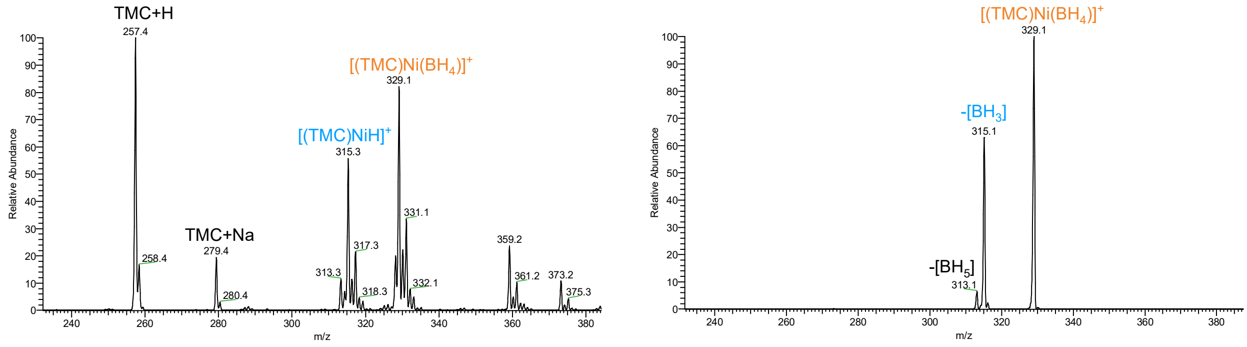

Figure S25. ESI-MS spectrum of a mixture of nickel complex and sodium borohydride (left). CID spectrum of mass-selected [(TMC)Ni(BH$_4$)]$^+$ (right).

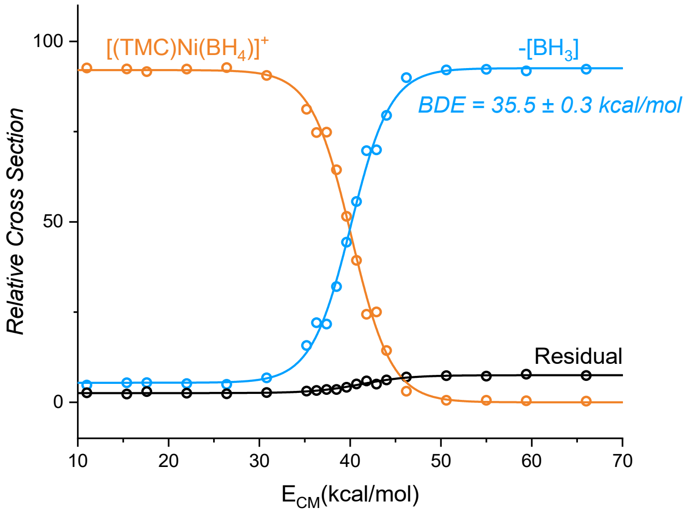

Figure S26. Breakdown diagram of [(TMC)Ni(BH$_4$)]$^+$, the dots are experimental data, the lines are fitted sigmoid functions. Linear extrapolation gives us the appearance energy (AE) or bond dissociation energy (BDE) of the fragmentation.

[(Tpy)Fe(BH$_4$)]$^+$:

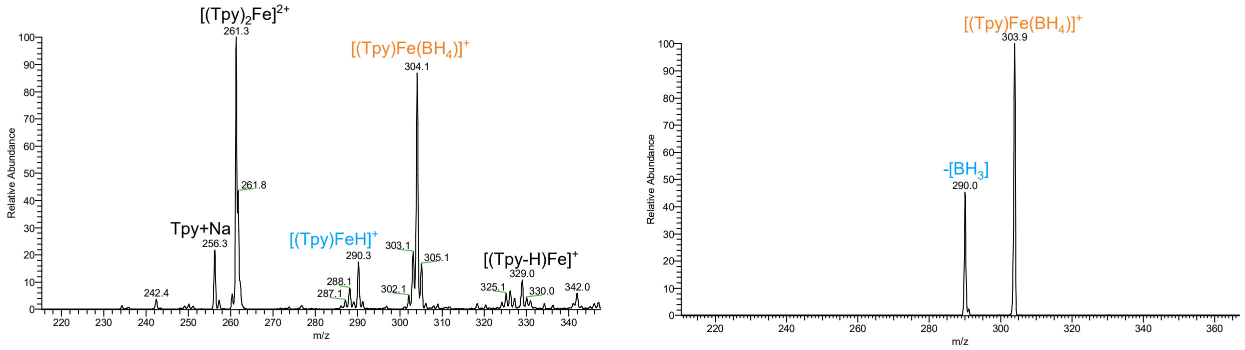

Figure S27. ESI-MS spectrum of a mixture of iron complex and sodium borohydride (left). CID spectrum of mass-selected [(Tpy)Fe(BH$_4$)]$^+$ (right).

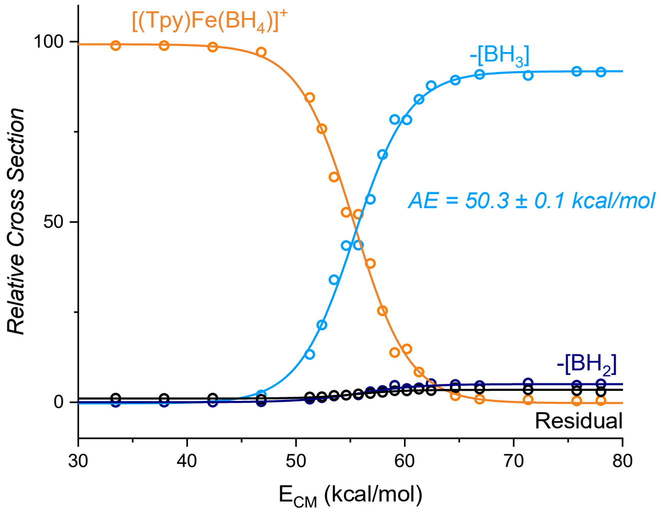

Figure S28. Breakdown diagram of [(Tpy)Fe(BH$_4$)]$^+$, the dots are experimental data, the lines are fitted sigmoid functions. Linear extrapolation gives us the appearance energy (AE) or bond dissociation energy (BDE) of the fragmentation.

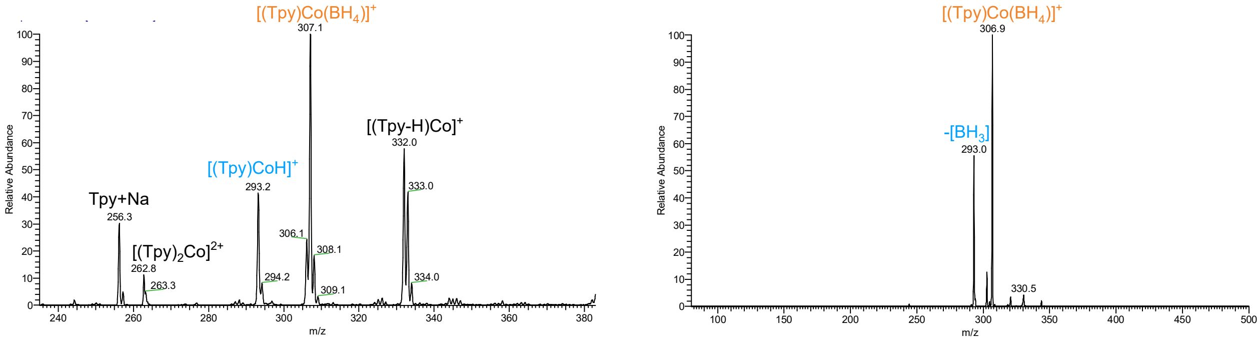

Figure S29. ESI-MS spectrum of a mixture of cobalt complex and sodium borohydride (left). CID spectrum of mass-selected [(Tpy)Co(BH$_4$)]$^+$ (right).

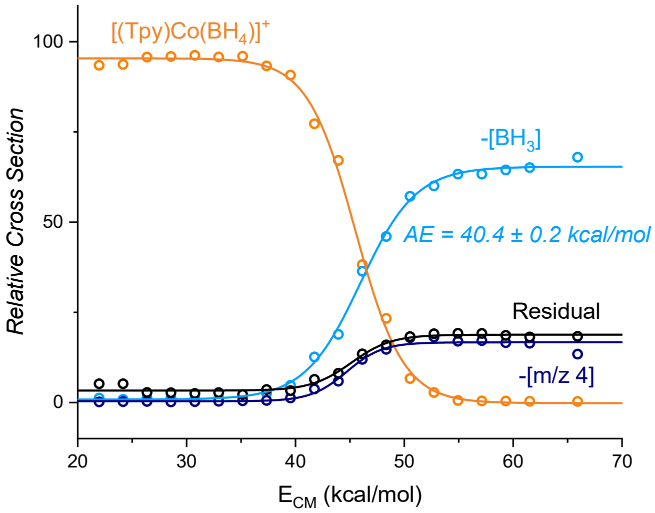

Figure S30. Breakdown diagram of [(Tpy)Co(BH_4)]$^+$, the dots are experimental data, the lines are fitted sigmoid functions. Linear extrapolation gives us the appearance energy (AE) or bond dissociation energy (BDE) of the fragmentation.

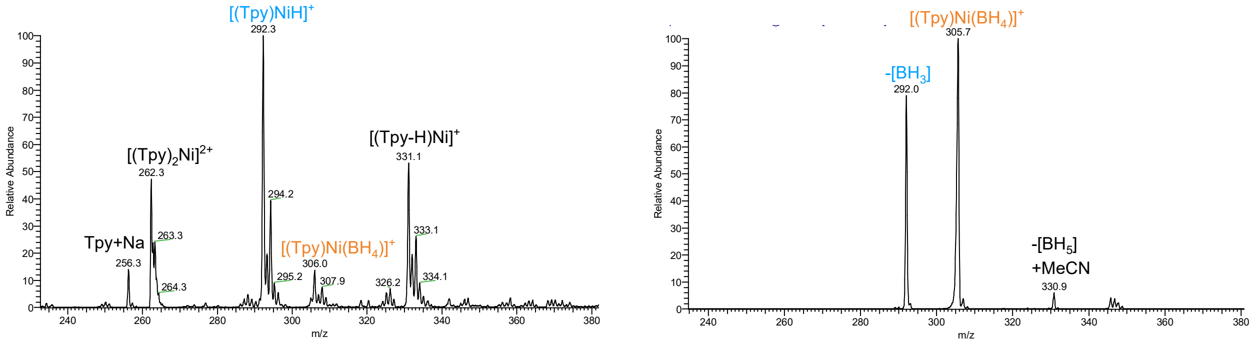

Figure S31. ESI-MS spectrum of a mixture of nickel complex and sodium borohydride (left). CID spectrum of mass-selected [(Tpy)Ni(BH_4)]$^+$ (right).

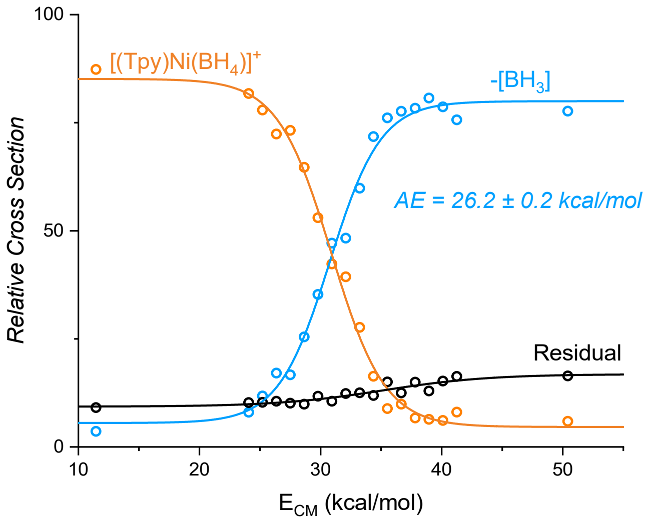

Figure S32. Breakdown diagram of [(Tpy)Ni(BH_4)]$^+$, the dots are experimental data, the lines are fitted sigmoid functions. Linear extrapolation gives us the appearance energy (AE) or bond dissociation energy (BDE) of the fragmentation.

Spectra and breakdown diagrams: P-based ligands

$[(dppe)_2Fe(BH_4)]^+$:

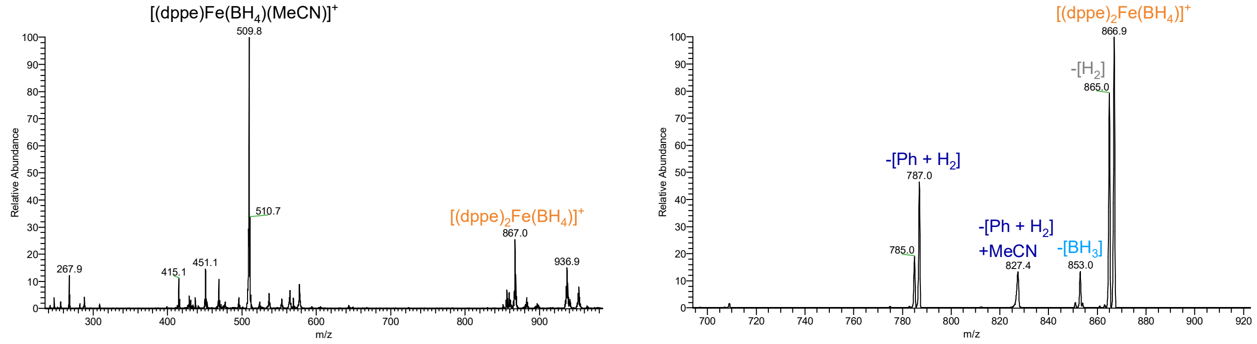

Figure S33. ESI-MS spectrum of a mixture of iron complex and sodium borohydride (left). CID spectrum of mass-selected $[(dppe)_2Fe(BH_4)]^+$ (right).

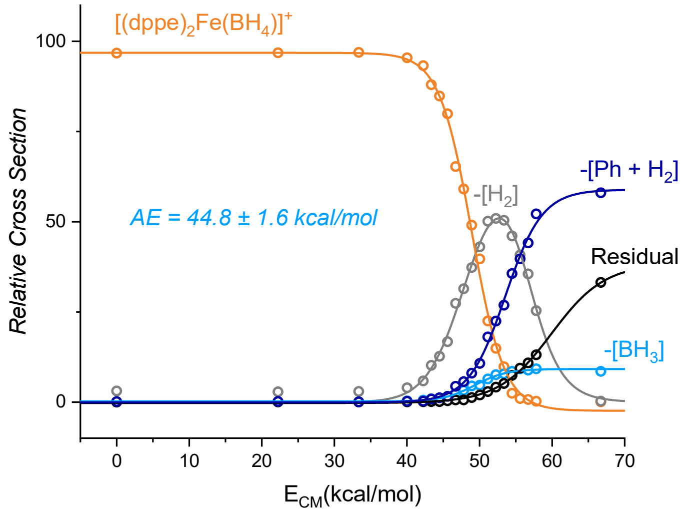

Figure S34. Breakdown diagram of $[(dppe)_2Fe(BH_4)]^+$, the dots are experimental data, the lines are fitted sigmoid functions. Linear extrapolation gives us the appearance energy (AE) or bond dissociation energy (BDE) of the fragmentation.

$[(dppe)_2Co(BH_4)]^+$:

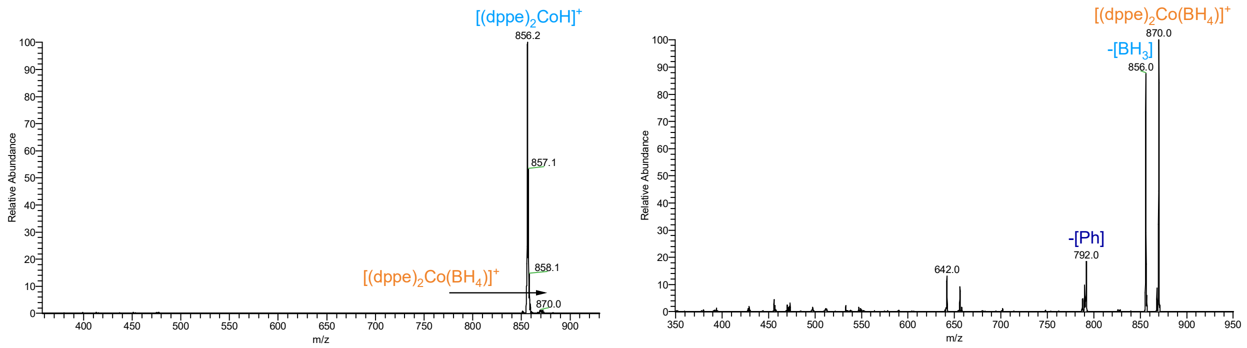

Figure S35. ESI-MS spectrum of a mixture of cobalt complex and sodium borohydride (left). CID spectrum of mass-selected $[(dppe)_2Co(BH_4)]^+$ (right).

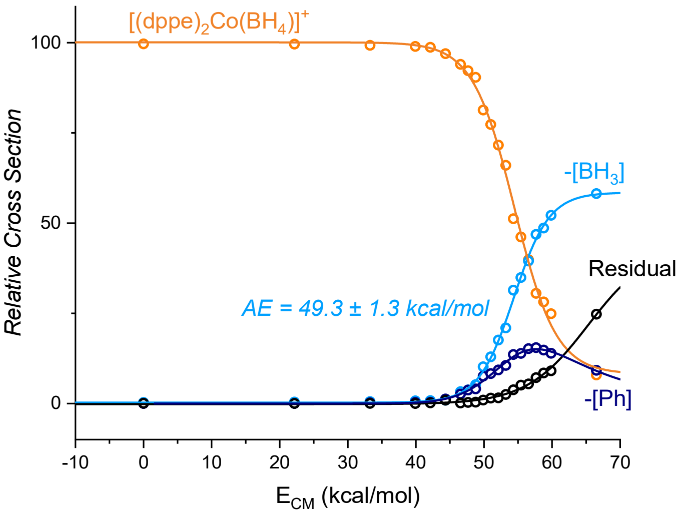

Figure S36. Breakdown diagram of [(dppe)$_2$Co(BH$_4$)]$^+$, the dots are experimental data, the lines are fitted sigmoid functions. Linear extrapolation gives us the appearance energy (AE) or bond dissociation energy (BDE) of the fragmentation.

[(dmpe)$_2$Fe(BH$_4$)]$^+$:

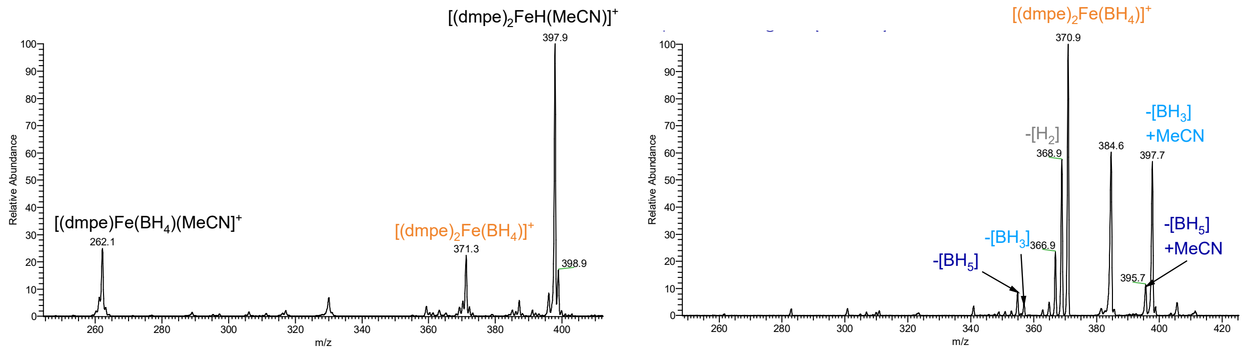

Figure S37. ESI-MS spectrum of a mixture of iron complex and sodium borohydride (left). CID spectrum of mass-selected [(dmpe)$_2$Fe(BH$_4$)]$^+$ (right).

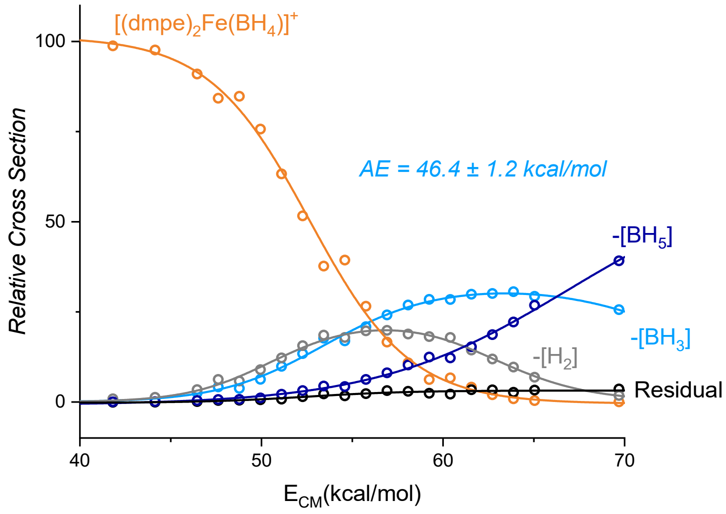

Figure S38. Breakdown diagram of [(dmpe)$_2$Fe(BH$_4$)]$^+$, the dots are experimental data, the lines are fitted sigmoid functions. Linear extrapolation gives us the appearance energy (AE) or bond dissociation energy (BDE) of the fragmentation.

$[(dppp)_2Fe(BH_4)]^+$:

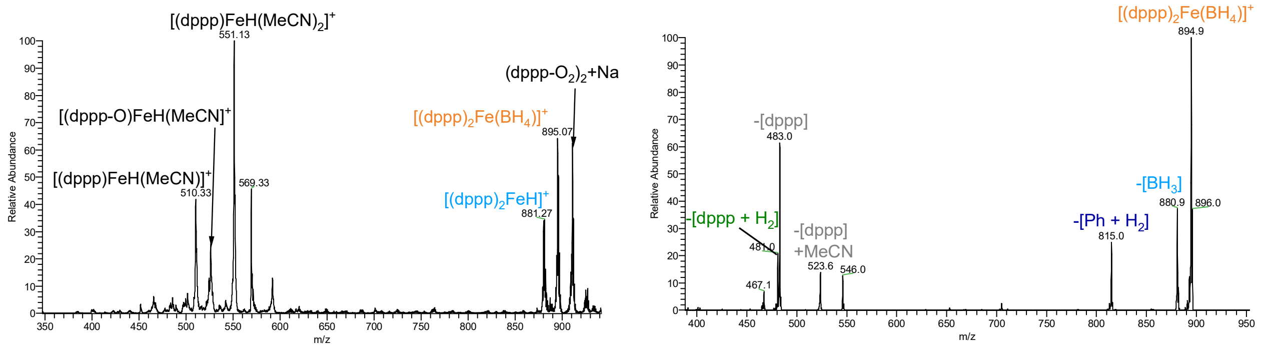

Figure S39. ESI-MS spectrum of a mixture of iron complex and sodium borohydride (left). CID spectrum of mass-selected $[(dppp)_2Fe(BH_4)]^+$ (right).

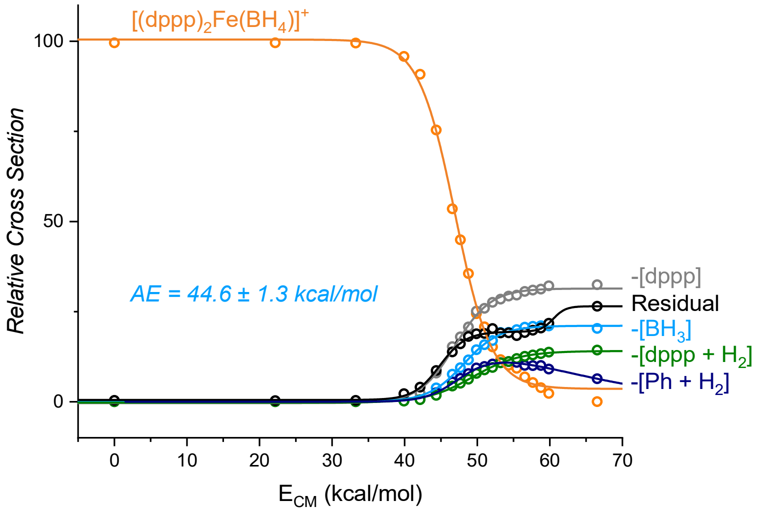

Figure S40. Breakdown diagram of $[(dppp)_2Fe(BH_4)]^+$, the dots are experimental data, the lines are fitted sigmoid functions. Linear extrapolation gives us the appearance energy (AE) or bond dissociation energy (BDE) of the fragmentation.

$[(dppp)_2Co(BH_4)]^+$:

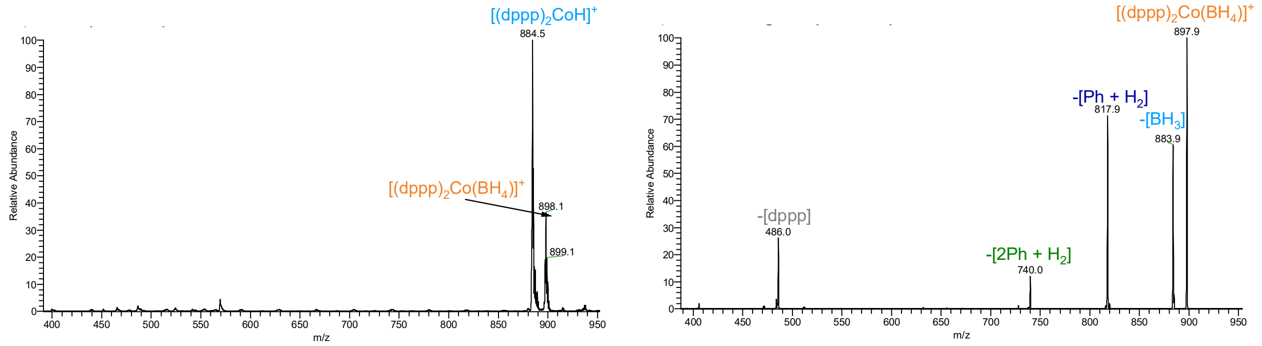

Figure S41. ESI-MS spectrum of a mixture of cobalt complex and sodium borohydride (left). CID spectrum of mass-selected $[(dppp)_2Co(BH_4)]^+$ (right).

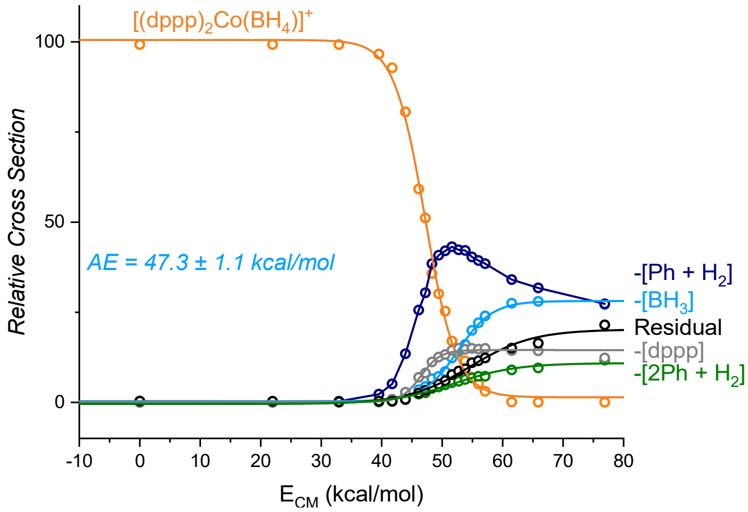

Figure S42. Breakdown diagram of [(dppp)$_2$Co(BH$_4$)]$^+$, the dots are experimental data, the lines are fitted sigmoid functions. Linear extrapolation gives us the appearance energy (AE) or bond dissociation energy (BDE) of the fragmentation.

[(dppp)$_2$Ni(BH$_4$)]$^+$:

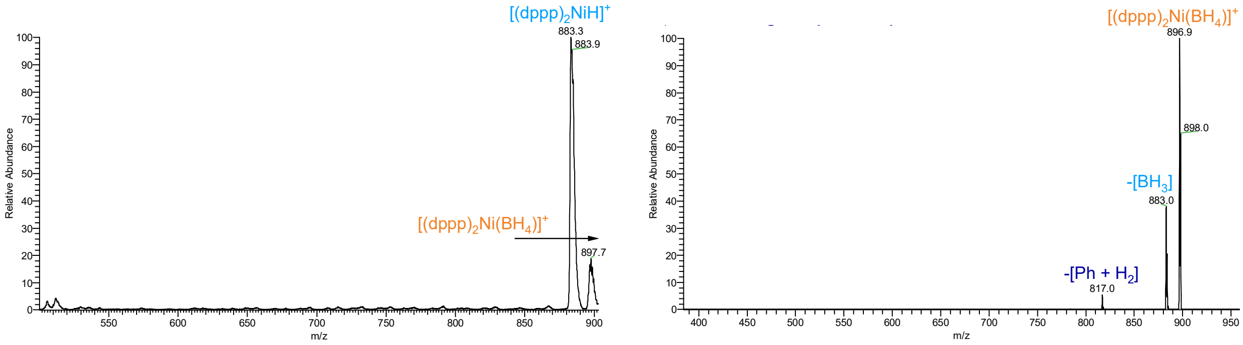

Figure S43. ESI-MS spectrum of a mixture of nickel complex and sodium borohydride (left). CID spectrum of mass-selected [(dppp)$_2$Ni(BH$_4$)]$^+$ (right).

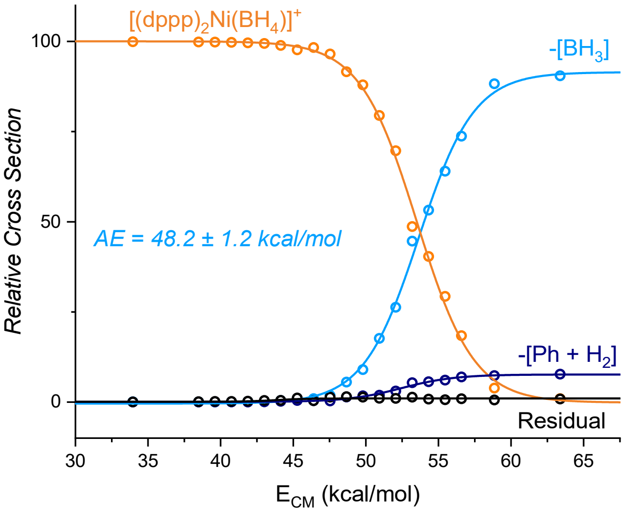

Figure S44. Breakdown diagram of [(dppp)$_2$Ni(BH$_4$)]$^+$, the dots are experimental data, the lines are fitted sigmoid functions. Linear extrapolation gives us the appearance energy (AE) or bond dissociation energy (BDE) of the fragmentation.

[(dppb)$_2$Fe(BH_4)]$^+$:

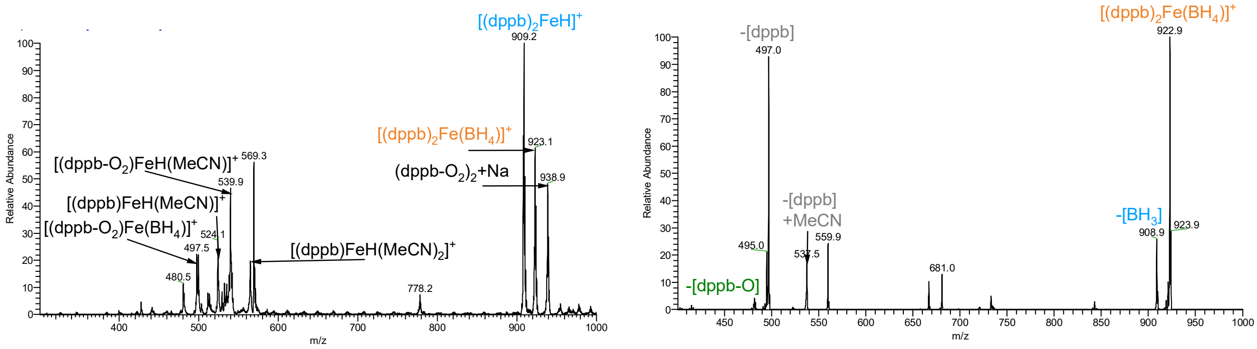

Figure S45. ESI-MS spectrum of a mixture of iron complex and sodium borohydride (left). CID spectrum of mass-selected [(dppb)$_2$Fe(BH_4)]$^+$ (right).

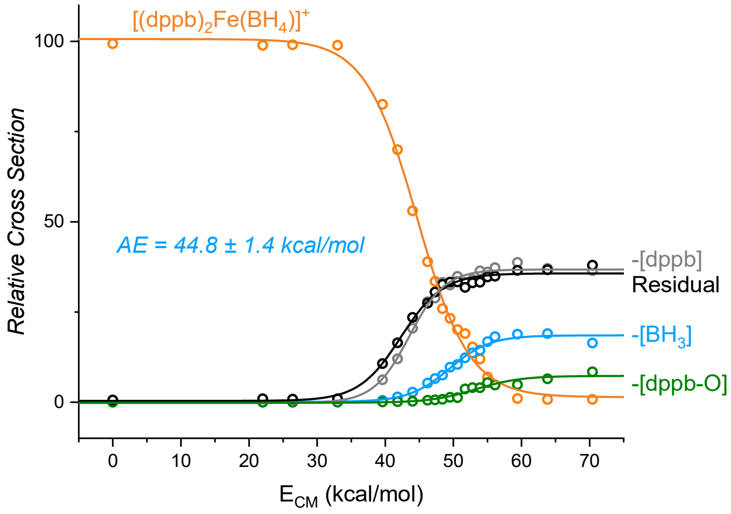

Figure S46. Breakdown diagram of [(dppb)$_2$Fe(BH_4)]$^+$, the dots are experimental data; the lines are fitted sigmoid functions. Linear extrapolation gives us the appearance energy (AE) or bond dissociation energy (BDE) of the fragmentation. Fragmentation leading to loss of oxidized ligand (green) is observed due to the isobaric mass of [(dppb)$_2$Fe(BH_4)]$^+$ (calc. m/z = 923.31) and [(dppb-O)(dppb-O_2)Na]$^+$ (calc. m/z = 923.32).

[(dppb)$_2$Co(BH_4)]$^+$:

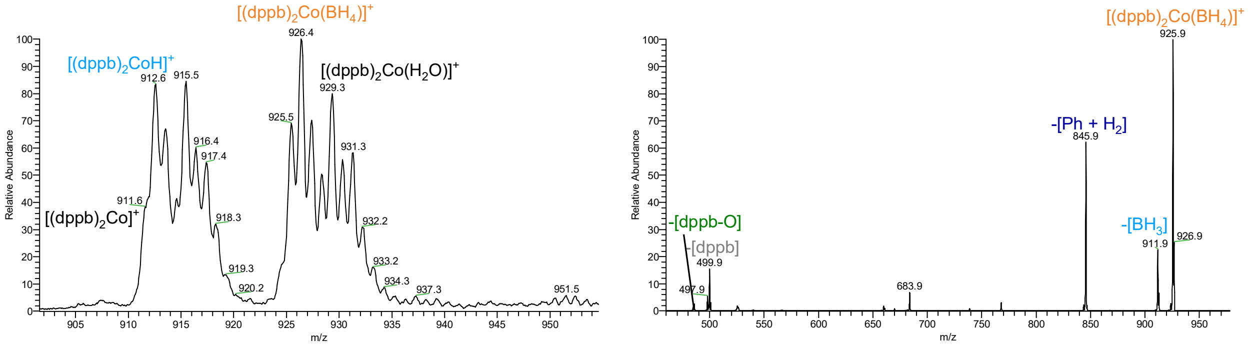

Figure S47. ESI-MS spectrum of a mixture of cobalt complex and sodium borohydride (left). CID spectrum of mass-selected [(dppb)$_2$Co(BH_4)]$^+$ (right).

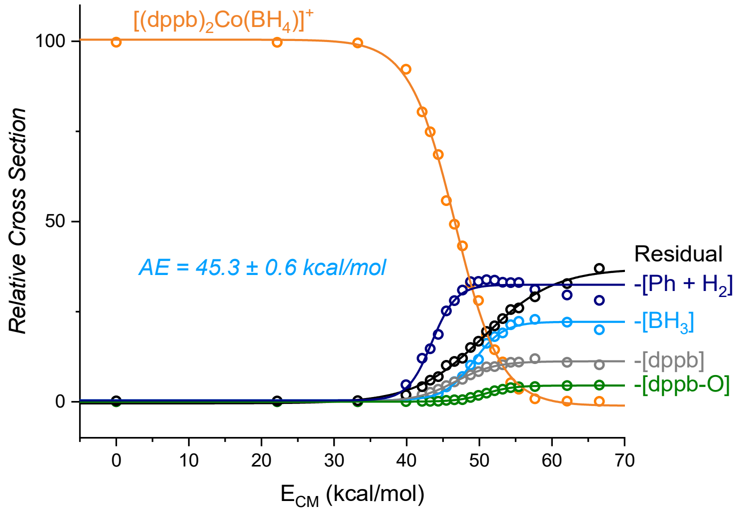

Figure S48. Breakdown diagram of [(dppb)$_2$Co(BH$_4$)]$^+$, the dots are experimental data, the lines are fitted sigmoid functions. Linear extrapolation gives us the appearance energy (AE) or bond dissociation energy (BDE) of the fragmentation.

[(dppb)$_2$Ni(BH$_4$)]$^+$:

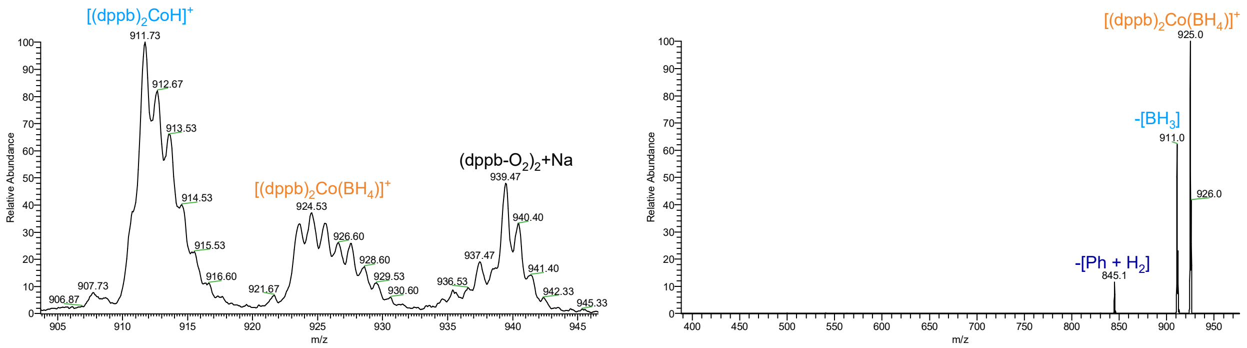

Figure S49. ESI-MS spectrum of a mixture of nickel complex and sodium borohydride (left). CID spectrum of mass-selected [(dppb)$_2$Ni(BH$_4$)]$^+$ (right).

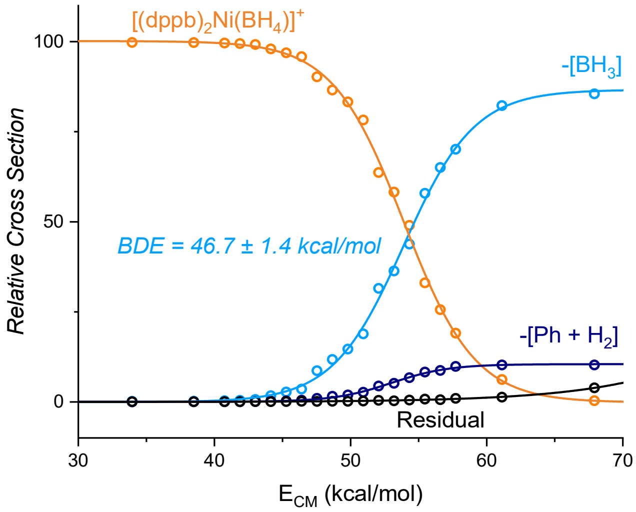

Figure S50. Breakdown diagram of $[(dppb)_2Ni(BH_4)]^+$, the dots are experimental data, the lines are fitted sigmoid functions. Linear extrapolation gives us the appearance energy (AE) or bond dissociation energy (BDE) of the fragmentation.

Results

Table S2. Summary of the obtained ΔE_{CID} corresponding to the loss of BH_3 from a common metal borohydride precursor.

N-based ligands	ΔE_{CID} (kcal/mol)	P-based ligands	ΔE_{CID} (kcal/mol)
$[(TPA)Fe(BH_4)]^+$	48.6 ± 0.9	$[(dppe)_2Fe(BH_4)]^+$	44.8 ± 1.6
$[(TPA)Co(BH_4)]^+$	42.6 ± 1.4	$[(dppe)_2Co(BH_4)]^+$	49.3 ± 1.3
$[(TPA)Ni(BH_4)]^+$	40.7 ± 0.08	$[(dmpe)_2Fe(BH_4)]^+$	46.4 ± 1.2
$[(TMC)Fe(BH_4)]^+$	45.3 ± 0.4	$[(dppp)_2Fe(BH_4)]^+$	44.6 ± 1.3
$[(TMC)Co(BH_4)]^+$	41.2 ± 0.1	$[(dppp)_2Co(BH_4)]^+$	47.3 ± 1.1
$[(TMC)Ni(BH_4)]^+$	35.5 ± 0.3	$[(dppp)_2Ni(BH_4)]^+$	48.2 ± 1.2
$[(Tpy)Fe(BH_4)]^+$	50.3 ± 0.1	$[(dppb)_2Fe(BH_4)]^+$	44.8 ± 1.4
$[(Tpy)Co(BH_4)]^+$	40.4 ± 0.2	$[(dppb)_2Co(BH_4)]^+$	45.3 ± 0.6
$[(Tpy)Ni(BH_4)]^+$	26.2 ± 0.2	$[(dppb)_2Ni(BH_4)]^+$	46.7 ± 1.4

Gas phase reactivity studies

Experimental details

The metal hydride anion complexes were generated analogously to the method described in the first section. The reactivity with thiophenol or formic acid was measured at 3-4 different pressures between 0.05 – 0.45 mTorr. Once stable pressure was acquired in the collision cell, the signal was recorded for at least 1 minute. In some cases, longer scan times were required to gain sufficient signal-to-noise ratio; each experiment was performed in triplo. The reactivity of $[(TPA)FeH]^{+}$ was investigated with a number of other neutral reactants. Ion-molecule reactions were observed for propionic acid and ethane thiol however the product ion signal was too low to quantify. No reaction was observed for isopropyl alcohol, pyridine or aniline as reactant gasses.

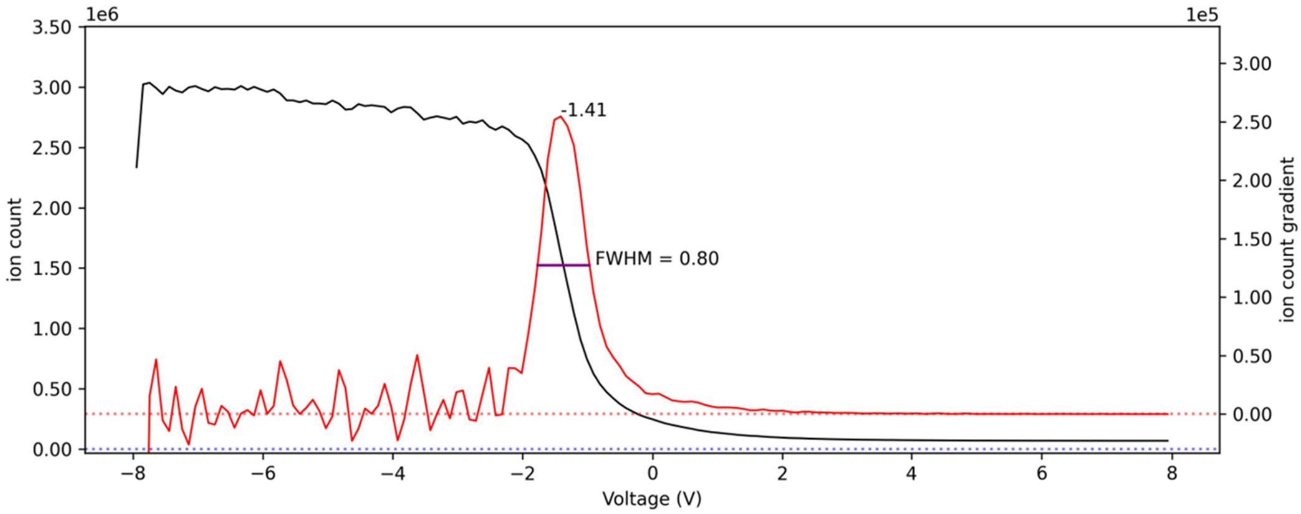

Figure S51. Retarded potential analysis to measure the ion kinetic energy distribution for $[(TPA)FeH]^{+}$. For all measured ions, the full width at half maximum (FWHM) of the measured distribution was in the range 0.7 to 0.9 eV. Depicted is the ion current intensity versus octopole voltage (black) and the corresponding derivative (red). In this case, the potential offset of the octopole was set to -1.41 V to achieve E_{LAB} = 0 eV.

Gas phase reactivity mass spectra

$[(TPA)FeH]^{+}$:

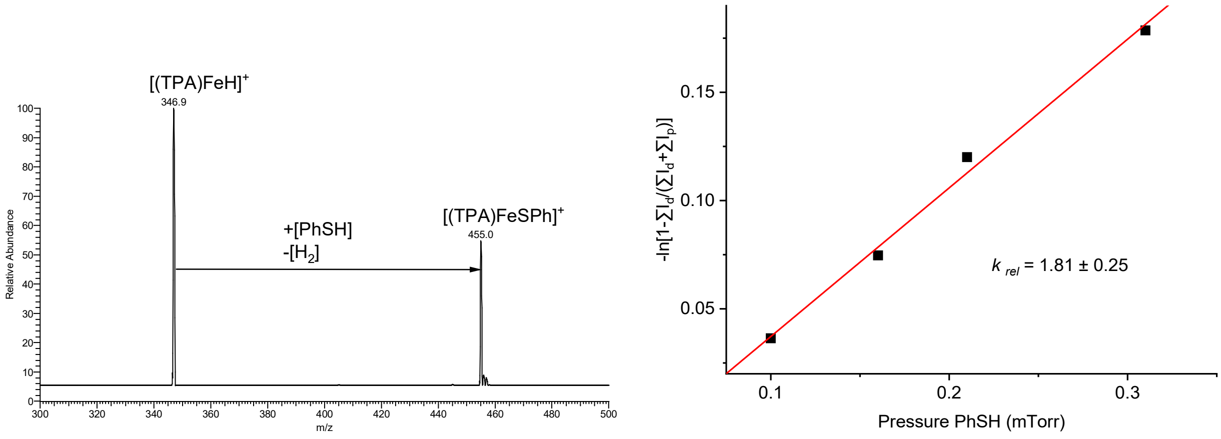

Figure S52. ESI-MS/MS spectrum of $[(TPA)FeH]^{+}$ with thiophenol reactant gas at 0.31 mTorr (left). On the right, a plot of the relative cross section of the product ion with respect to the precursor ion versus the reactant gas pressure. In red is the linear fit of the cross-section pressure dependence; the slope gives a relative rate constant for the reaction.

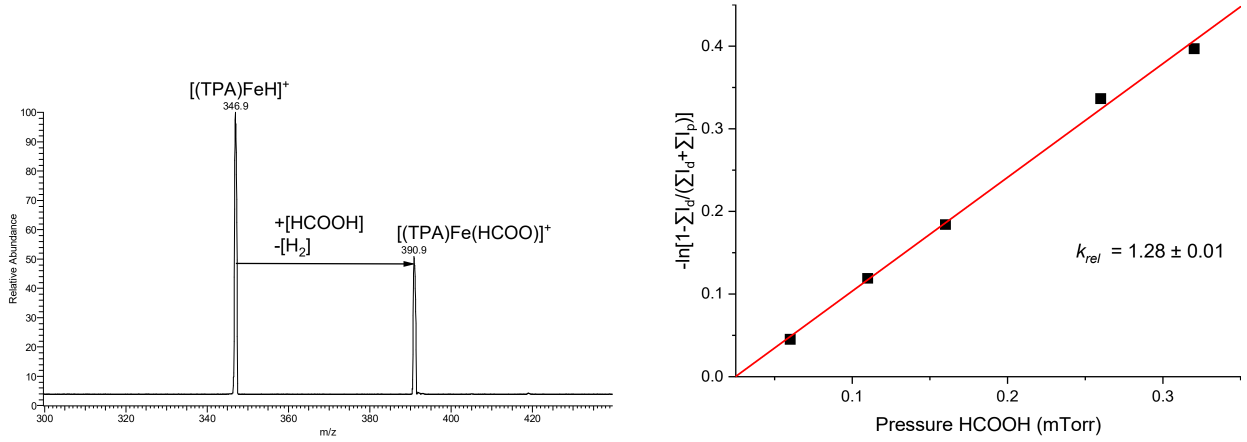

Figure S53. ESI-MS/MS spectrum of $[(TPA)FeH]^+$ with formic acid reactant gas at 0.32 mTorr (left). On the right, a plot of the relative cross section of the product ion with respect to the precursor ion versus the reactant gas pressure. In red is the linear fit of the cross-section pressure dependence; the slope gives a relative rate constant for the reaction.

$[(TPA)CoH]^+$:

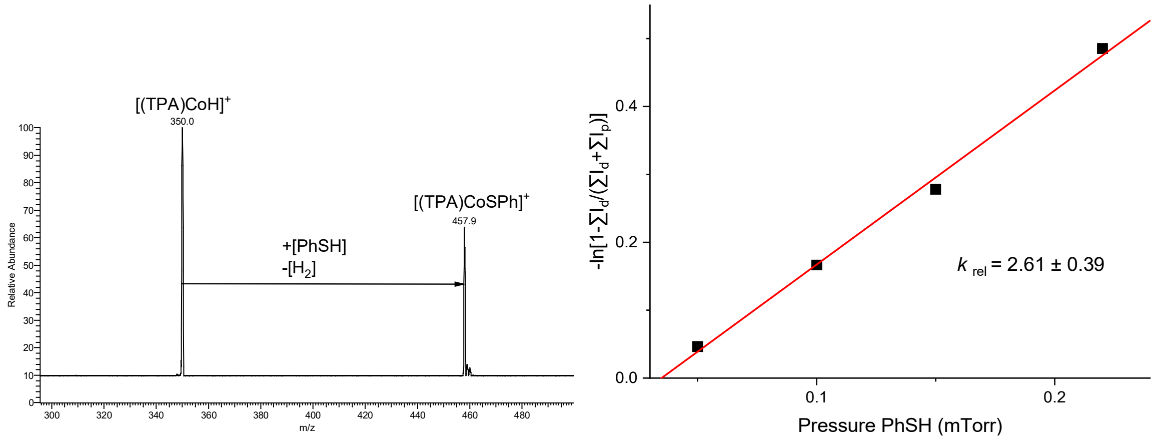

Figure S54. ESI-MS/MS spectrum of $[(TPA)CoH]^+$ with thiophenol reactant gas at 0.22 mTorr (left). On the right, a plot of the relative cross section of the product ion with respect to the precursor ion versus the reactant gas pressure. In red is the linear fit of the cross-section pressure dependence; the slope gives a relative rate constant for the reaction.

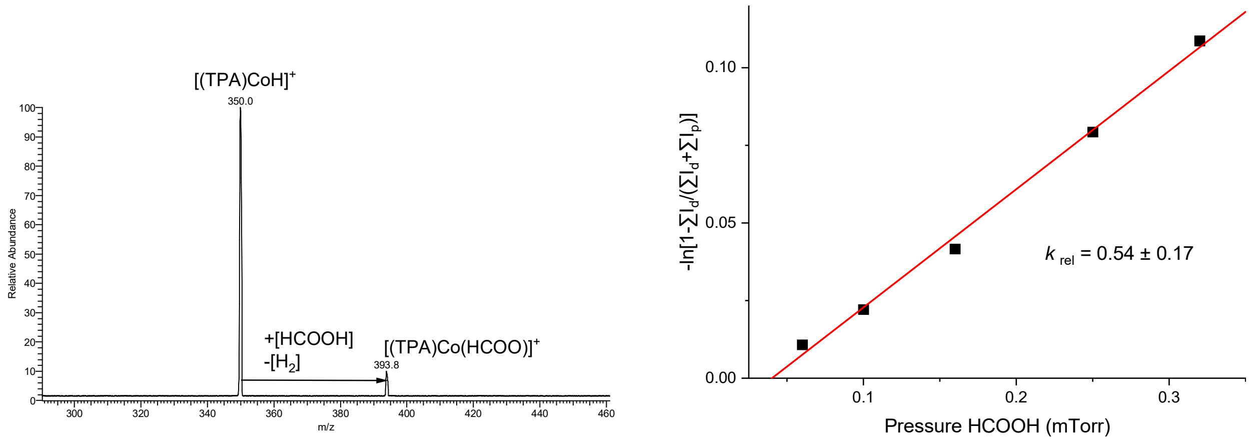

Figure S55. ESI-MS/MS spectrum of $[(TPA)CoH]^+$ with formic acid reactant gas at 0.25 mTorr (left). On the right, a plot of the relative cross section of the product ion with respect to the precursor ion versus the reactant gas pressure. In red is the linear fit of the cross-section pressure dependence; the slope gives a relative rate constant for the reaction.

[(TPA)NiH]$^+$:

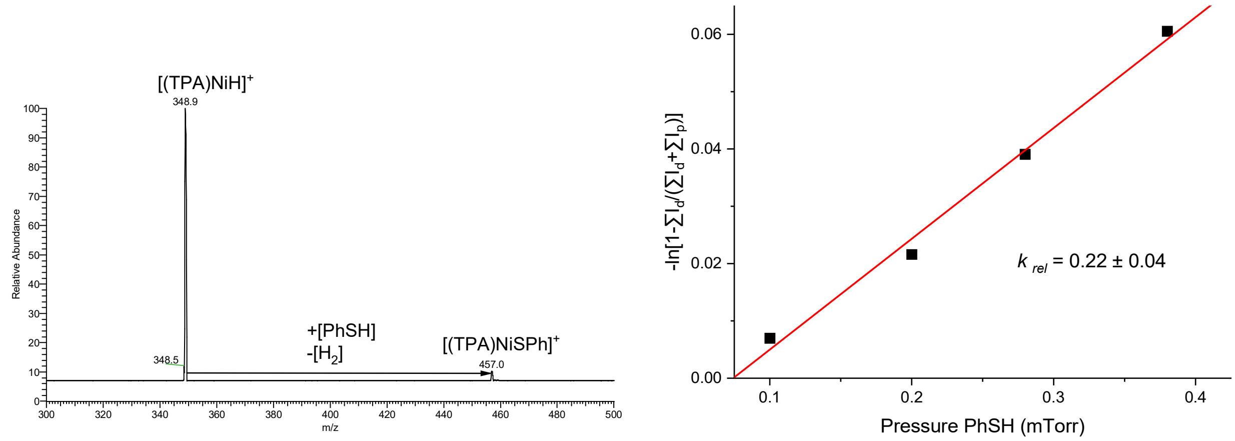

Figure S56. ESI-MS/MS spectrum of [(TPA)NiH]$^+$ with thiophenol reactant gas at 0.28 mTorr (left). On the right, a plot of the relative cross section of the product ion with respect to the precursor ion versus the reactant gas pressure. In red is the linear fit of the cross-section pressure dependence; the slope gives a relative rate constant for the reaction.

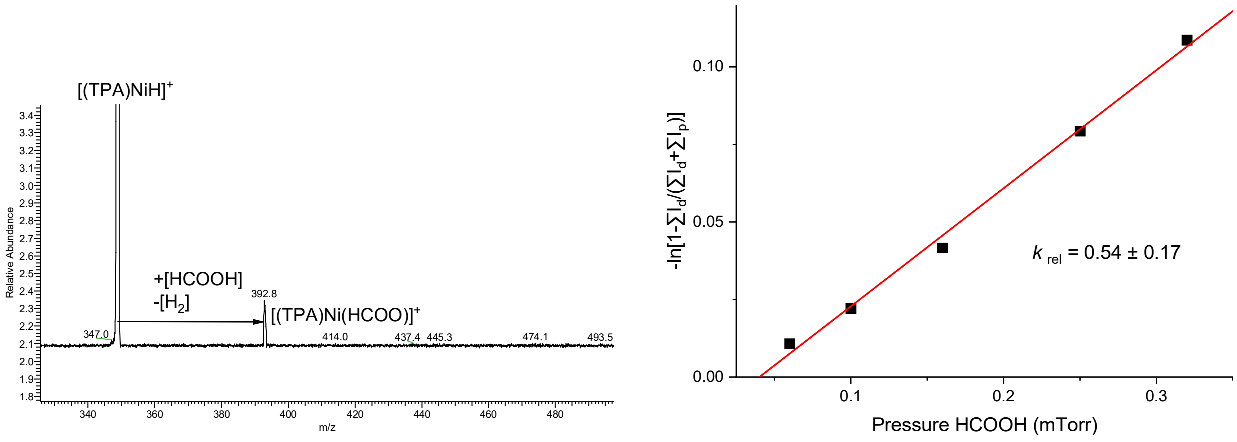

Figure S57. ESI-MS/MS spectrum of [(TPA)NiH]$^+$ with formic acid reactant gas at 0.41 mTorr (left). On the right, a plot of the relative cross section of the product ion with respect to the precursor ion versus the reactant gas pressure. In red is the linear fit of the cross-section pressure dependence; the slope gives a relative rate constant for the reaction.

[(Tpy)FeH]$^+$:

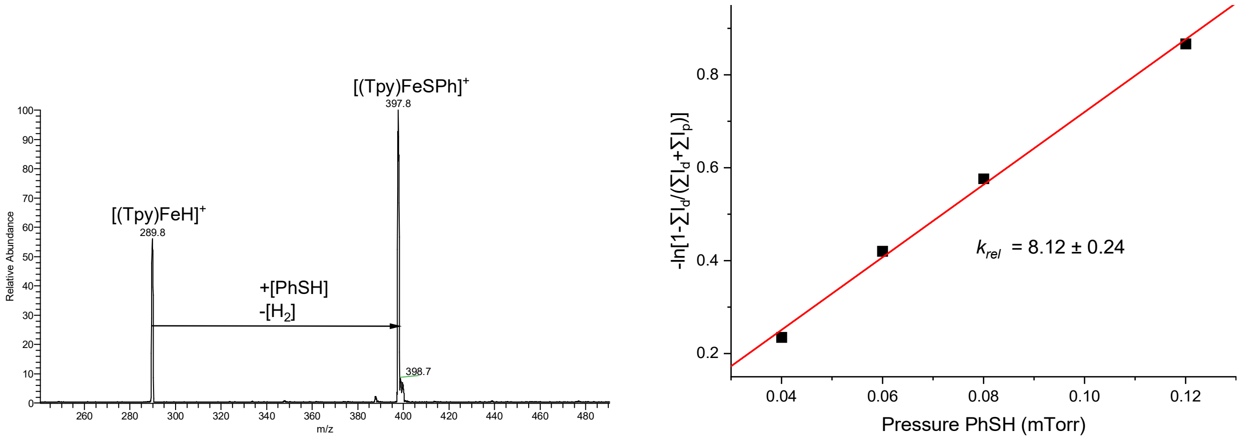

Figure S58. ESI-MS/MS spectrum of [(Tpy)FeH]$^+$ with thiophenol reactant gas at 0.12 mTorr (left). On the right, a plot of the relative cross section of the product ion with respect to the precursor ion versus the reactant gas pressure. In red is the linear fit of the cross-section pressure dependence; the slope gives a relative rate constant for the reaction.

[(Tpy)CoH]⁺:

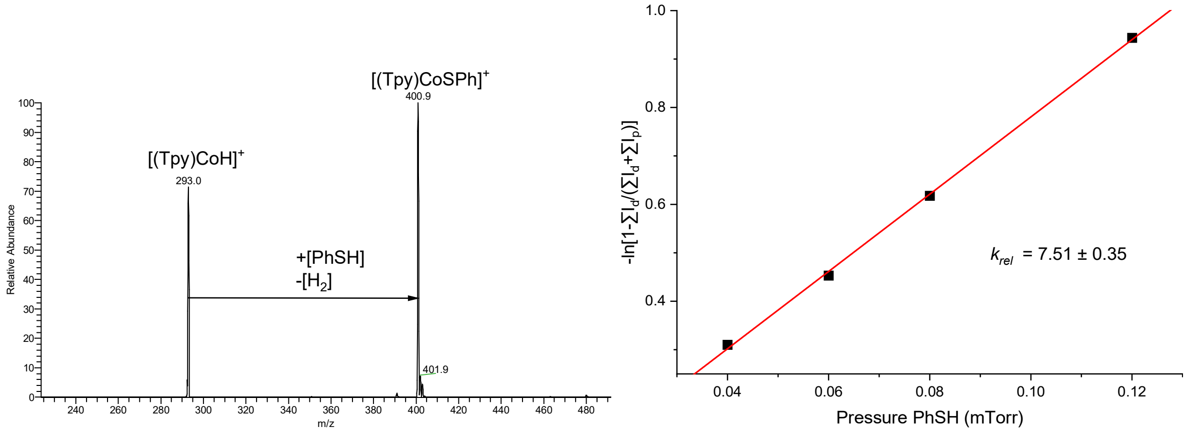

Figure S59. ESI-MS/MS spectrum of $[(Tpy)CoH]^+$ with thiophenol reactant gas at 0.12 mTorr (left). On the right, a plot of the relative cross section of the product ion with respect to the precursor ion versus the reactant gas pressure. In red is the linear fit of the cross-section pressure dependence; the slope gives a relative rate constant for the reaction.

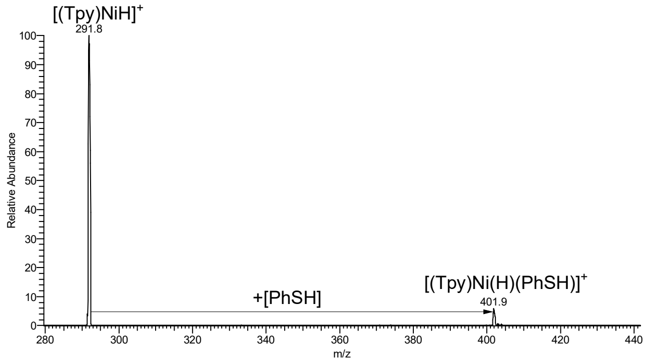

Figure S60. ESI-MS/MS spectrum of $[(Tpy)NiH]^+$ with thiophenol reactant gas at 0.3 mTorr.

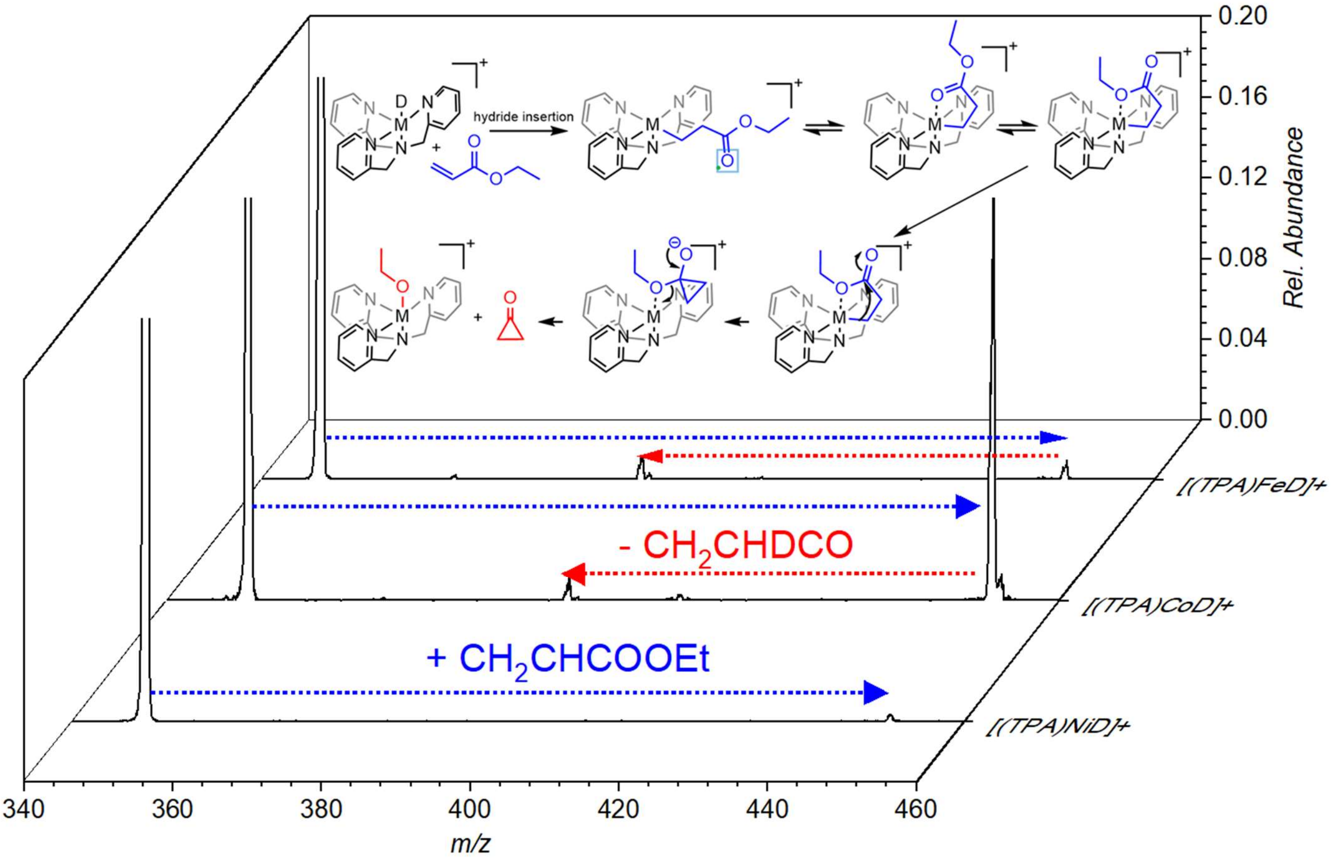

Figure S61. Gas-phase reactivity of mass selected ions $[(TPA)MD]^+$ in collisions with ethyl acrylate at 0.24 mTorr of pressure and zero collision energy. All complexes react with ethyl acrylate by formation of an adduct (blue arrow). We cannot distinguish, whether this reaction is a pure adduct formation or the insertion reaction. However, the rate for the product formation increases in the order Ni<Fe<Co. Hence, it follows the same reactivity order as the reactions with thiophenol and may thus correspond to the 1,2-insertion reaction. In addition, the reaction of the Fe and Co complexes also leads to the formation of $[(TPA)M(OEt)]^+$. This reactivity could be explained by the initial insertion reaction followed by the elimination of cyclopropanone. The rate of this reactivity channel follows the order Ni<<Co<Fe.

Results

Table S3. Summary of the obtained kinetic for the gas phase reactivity of the metal hydrides with various substrates

Metal hydride complex	Relative rate constant *k* with thiophenol	Relative rate constant *k* with formic acid
$[(TPA)FeH]^+$	1.81 ± 0.25	1.28 ± 0.01
$[(TPA)CoH]^+$	2.61 ± 0.39	0.54 ± 0.17
$[(TPA)NiH]^+$	0.22 ± 0.04	0.01 ± 0.0
$[(Tpy)FeH]^+$	8.12 ± 0.24	-
$[(Tpy)CoH]^+$	7.51 ± 0.35	-

Density Functional Theory studies

Potential energy surfaces and structures

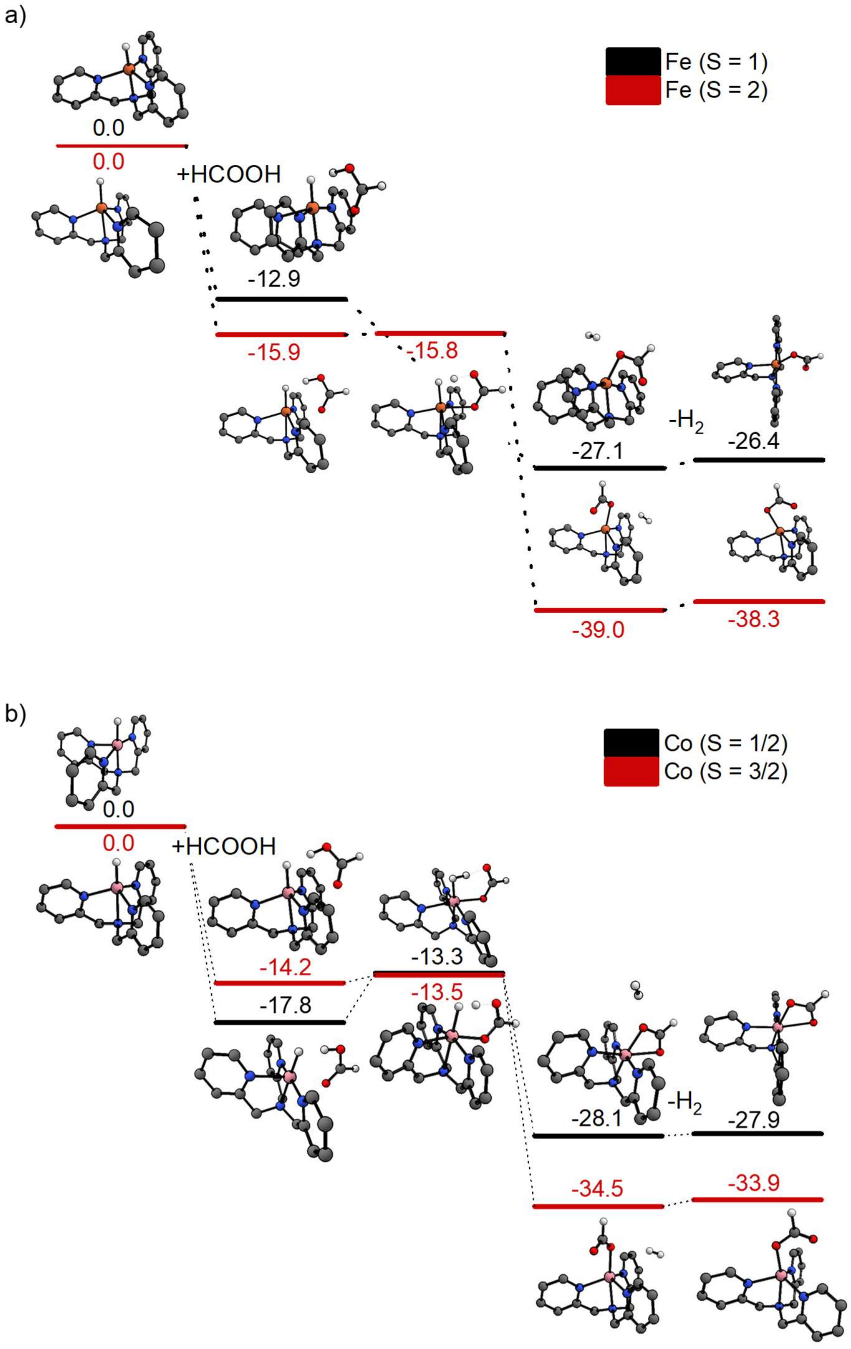

Figure S62. a) Potential energy surface ($\Delta\Delta H^{0K}$, in kcal/mol, B3LYP-D3/6-311++G(d,p)) of $[(TPA)FeH]^+$ with formic acid for the S=1 (black) and the S = 2 spin state (red). b) Potential energy surface ($\Delta\Delta H^{0K}$, in kcal/mol, B3LYP-D3/6-311++G(d,p)) of $[(TPA)CoH]^+$ with formic acid for the S=1/2 (black) and the S = 3/2 spin state (red). *Calculated structures are depicted above or below the energy bar. Hydrogen atoms of the C-H bonds of the ligand were removed for clarity.

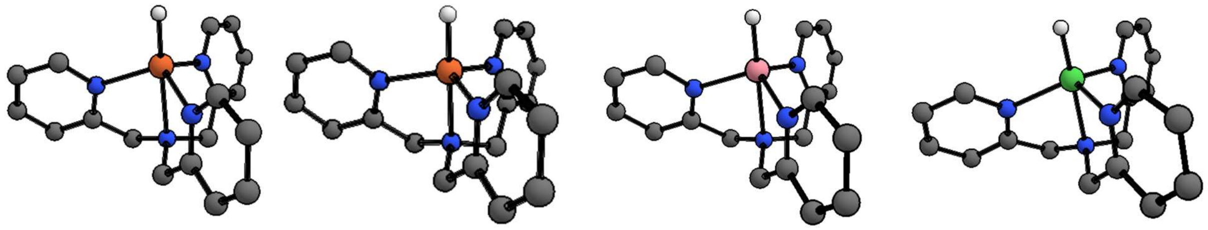

Figure S63. High spin metal complexes with the general formula $[(TPA)MH]^{+}$. From left to right: Fe^{II} (S=2), Fe^{II} (S=1), Co^{II} (S=3/2), Ni^{II} (S=1). Displayed structures calculated at the B3LYP-D3/6-311++G(d,p) level of theory, hydrogen atoms on carbons removed for clarity.

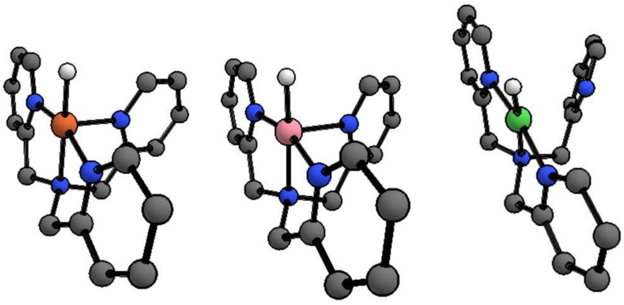

Figure S64. Low spin metal complexes with the general formula $[(TPA)MH]^{+}$. From left to right: Fe^{II} (S=0), Co^{II} (S=1/2), Ni^{II} (S=0). Displayed structures calculated at the B3LYP-D3/6-311++G(d,p) level of theory, hydrogen atoms on carbons removed for clarity.

Results

Table S4. Gas phase hydricities $\Delta E^{Hydricity}$ calculcated for complexes with the general formula $[(L^{N})MH]^{+}$ according to equation 1 in the main text. The value for $\Delta E^{Hydricity}([(BH_4)]^{+})$ is calculated at B3LYP/6-311++G(d,p). $\Delta E^{Coulomb}([(L)M^{II}(BH_4)]^{+})$ is taken from table S8.

**Equation 1**		$[(TPA)MH]^{+}$		$[(tPy)MH]^{+}$		$[(TMC)MH]^{+}$	
	$\Delta E^{Hydricity}$ $([(BH_4)]^{+})$ (kcal/mol)	$\Delta E^{Coulomb}$ $([(L)M^{II}(BH_4)]^{+})$ From table S8 (kcal/mol)	$\Delta E^{Hydricity}$ $([(L)M^{II}H]^{+})$ (kcal/mol)	$\Delta E^{Coulomb}$ $([(L)M^{II}(BH_4)]^{+})$ From table S8 (kcal/mol)	$\Delta E^{Hydricity}$ $([(L)M^{II}H]^{+})$	$\Delta E^{Coulomb}$ $([(L)M^{II}(BH_4)]^{+})$ From table S8 (kcal/mol)	$\Delta E^{Hydricity}$ $([(L)M^{II}H]^{+})$
Fe	71.0	187.2	209.6	219.0	239.7	201.5	227.2
Co	71.0	189.9	218.3	222.3	252.9	192.2	222.0
Ni	71.0	195.3	225.6	216.6	261.4	159.3	194.8

Table S5. Relative energies in kcal/mol calculated at B3LYP-D3/6-311++G(d,p) of the high spin and low spin complexes with the general formula $[(L^N)MH]^+$. Energies are relative to energetically the most favoured spin state for each complex. In bold are the spin states as determined by IRPD analysis.

	$[(TPA)MH]^+$	$[(tPy)MH]^+$	$[(TMC)MH]^+$
Fe (S = 0)	+17.9	+31.9	+17.8
Fe (S = 1)	+4.2	0	+13
Fe (S = 2)	**0**	**+11.5**	**0**
Co (S = 1/2)	**+3.3**	**0**	+5.4
Co (S = 3/2)	0	+19.4	**0**
Ni (S = 0)	**0**	**0**	**+12.5**
Ni (S = 1)	+0.7	+50.5	0

Table S6. Relative energies in kcal/mol calculated at B3LYP-D3/6-311++G(d,p) of the high spin and low spin complexes with general formula $[(L^P)MH]^+$. Energies are relative to energetically most favoured spin state for each complex.

	$[(dppe)_2MH]^+$	$[(dppp)_2MH]^+$	$[(dmpe)_2MH]^+$
Fe (S = 0)	0	+10.3	+1.5
Fe (S = 1)	+2.5	0	0
Fe (S = 2)	+11.6	+9.5	+16.2
Co (S = 1/2)	0	0	-
Co (S = 3/2)	+18.9	+17.0	-
Ni (S = 0)	0	0	-
Ni (S = 1)	+7.3	+19.3	-

Table S7. Relative endothermicities in the gas phase of the reaction $[(L^N)M(BH_4)]^+$ -> $[(L^N)M]^{2+}$ + BH_4^- in kcal/mol calculated at B3LYP-D3/6-311++G(d,p) examined for high and low spin metal complexes. Energies are relative to the $[(L^N)M(BH_4)]^+$ reactant ion.

	$[(TPA)M]^{2+}$	$[(tPy)M]^{2+}$	$[(TMC)M]^{2+}$
Fe (S = 0)	212.3	239.7	193.3
Fe (S = 1)	196.4	224.8	176.3
Fe (S = 2)	187.2	219.0	201.5
Co (S = 1/2)	189.9	222.3	172.4
Co (S = 3/2)	188.7	228.9	192.2
Ni (S = 0)	195.3	216.6	159.3
Ni (S = 1)	193.1	223.0	190.6

Table S8. Relative endothermicities in the gas phase of the reaction $[(L^P)M(BH_4)]^+$ -> $[(L^P)M]^{2+} + BH_4^-$ in kcal/mol calculated at B3LYP-D3/6-311++G(d,p) examined for high and low spin metal complexes. Energies are relative to the $[(L^P)M(BH_4)]^+$ reactant ion.

	$[(dppe)_2M]^{2+} + BH_4^-$	$[(dppp)_2M]^{2+} + BH_4^-$	$[(dmpe)_2M]^{2+} + BH_4^-$
Fe (S = 0)	171.2	167.6	198.7
Fe (S = 1)	153.7	156.5	174.6
Fe (S = 2)	165.9	152.1	181.1
Co (S = 1/2)	150.2	149.0	-
Co (S = 3/2)	158.1	144.6	-
Ni (S = 0)	138.4	137.2	-
Ni (S = 1)	160.3	144.5	-

Table S9. Relative endothermicities in the gas phase of the reaction $[(L^N)M(BH_4)]^+$ -> $[(L^N)MH]^+ + BH_3$ in kcal/mol calculated at B3LYP-D3/6-311++G(d,p) examined for high and low spin metal complexes. Energies are relative to the $[(L^N)M(BH_4)]^+$ reactant ion.

	$[(TPA)MH]^+$	$[(tPy)MH]^+$	$[(TMC)MH]^+$
Fe (S = 0)	52.9	59.0	23.5
Fe (S = 1)	26.3	37.4	34.3
Fe (S = 2)	38.0	55.6	36.9
Co (S = 1/2)	24.3	34.8	28.7
Co (S = 3/2)	36.0	50.8	36.4
Ni (S = 0)	18.3	22.5	22.0
Ni (S = 1)	39.9	76.1	29.4

Table S10. Relative endothermicities in the gas phase of the reaction $[(L^P)M(BH_4)]^+$ -> $[(L^P)MH]^+ + BH_3$ in kcal/mol calculated at B3LYP-D3/6-311++G(d,p) examined for high and low spin metal complexes. Energies are relative to the $[(L^P)M(BH_4)]^+$ reactant ion.

	$[(dppe)_2MH]^+$	$[(dppp)_2MH]^+$	$[(dmpe)_2MH]^+$
Fe (S = 0)	14.9	15.6	20.5
Fe (S = 1)	28.1	21.8	24.4
Fe (S = 2)	36.9	36.5	40.3
Co (S = 1/2)	24.0	10.9	-
Co (S = 3/2)	35.5	31.1	-
Ni (S = 0)	24.3	0.1	-
Ni (S = 1)	17.3	25.6	-

Table S11. Calculated average P-M bond lengths in Å for the hydride acceptor complexes $[(dppx)_2M]^{2+}$ with x = e, p, b and M = Fe, Co, Ni. To accept a hydride, the metal complex needs an empty d-orbital. This results in an electron configuration for Fe (S=0/1), Co (S=1/2), Ni (S=0). The S=1 electron configuration for Fe was calculated to be energetically more favoured.

	$[(dppe)_2M]^{2+}$	$[(dppp)_2M]^{2+}$	$[(dppb)_2M]^{2+}$
Fe	2.36	2.40	2.44
Co	2.31	2.35	2.37
Ni	2.29	2.31	2.33

Table S12. Calculated bite angle (BA) and twist angles (TA) for the hydride acceptor complexes $[(dppx)_2M]^{2+}$ with x = e, p, b and M = Fe, Co, Ni. To accept a hydride, the metal complex needs an empty d-orbital. This results in an electron configuration for Fe (S=0/1), Co (S=1/2), Ni (S=0). The S=1 electron configuration for Fe was calculated to be energetically more favoured.

	$[(dppe)_2M]^{2+}$		$[(dppp)_2M]^{2+}$		$[(dppb)_2M]^{2+}$	
	BA	TA	BA	TA	BA	TA
Fe	82.3	12.1	89.7	38.5	97.0	57.7
Co	84.8	4.4	88.9	38.2	93.1	42.7
Ni	85.1	14.7	90.8	39.6	94.1	51.2
Average BA/TA	84.1 ± 1.3	10.4 ± 4.4	89.8 ± 0.8	38.8 ± 0.6	94.7 ± 1.7	50.5 ± 6.2

Table S13. Calculated natural atomic charges on the metal center, hydride atom and on the metal hydride bond of the complexes with general formula $[(L^N)MH]^+$. The electron configuration was chosen based upon the IRPD studies as explained in the article.

	$[(TPA)MH]^+$			$[(tPy)MH]^+$			$[(TMC)MH]^+$		
	M	H	M-H	M	H	M-H	M	H	M-H
Fe	1.316	-0.527	0.789	1.404	-0.614	0.790	1.242	-0.466	0.776
Co	1.086	-0.373	0.713	1.364	-0.600	0.764	1.087	-0.392	0.695
Ni	0.892	-0.306	0.586	0.884	-0.316	0.568	0.910	-0.325	0.585

Table S14. Calculated natural atomic charges on the metal center, hydride atom and on the metal hydride bond of the complexes with general formula $[(L^P)MH]^+$. The most stable electron configuration was selected for the metal complexes: Fe (S=1), Co (S=1/2), Ni (S=0).

	$[(dppe)_2MH]^+$			$[(dppp)_2MH]^+$		
	M	H	M-H	M	H	M-H
Fe	1.192	-0.432	0.760	1.187	-0.368	0.819
Co	1.162	-0.449	0.713	1.185	-0.312	0.873
Ni	1.216	-0.430	0.786	1.148	-0.316	0.832
